# Coordinated P450–UGT detoxification contributes to furanocoumarin tolerance associated with host plant range divergence in *Helicoverpa*

**DOI:** 10.1371/journal.pbio.3004013

**Published:** 2026-09-22

**Authors:** Huidong Wang, Yajie Kong, Xinyue Su, Yu Shi, Jianpeng Zhang, Yinjia Wang, Yajuan Xiao, Xing Geng, Jing Song, Yueru Ye, Kairan Zuo, Chris Bass, Shutang Zhou

**Affiliations:** 1 State Key Laboratory of Cotton Bio-breeding and Integrated Utilization, School of Life Sciences, College of Agriculture, Henan University, Kaifeng, Henan, China; 2 State Key Laboratory of Agricultural and Forestry Biosecurity, Department of Entomology, College of Plant Protection, Nanjing Agricultural University, Nanjing, China; 3 School of Wetlands, Yancheng Teachers University, Yancheng, China; 4 Centre for Ecology and Conservation, University of Exeter, Penryn, United Kingdom; Cornell University, UNITED STATES OF AMERICA

## Abstract

Plants and herbivorous insects are engaged in a continuous evolutionary arms race driven by chemical defences and counter-defences. How insects integrate distinct detoxification systems to overcome diverse phytochemicals, and how such mechanisms contribute to host-range divergence, remains poorly understood. Here, we uncover a cooperative cytochrome P450–UDP-glycosyltransferase (UGT) cascade that mediates furanocoumarin tolerance in *Helicoverpa armigera*. Comparative genomic analyses across *Helicoverpa* and related noctuid species revealed that the UGT33 family is the most extensively expanded and dynamically diversified UGT lineage in *Helicoverpa*. Targeted CRISPR–Cas9 knockouts of UGT33 gene clusters in polyphagous *H. armigera* demonstrated their essential roles in detoxifying the furanocoumarins xanthotoxin and imperatorin. Combined metabolic and functional assays further established a sequential detoxification pathway, in which the cytochrome P450 CYP6AE19 catalyses either the O-dealkylation of xanthotoxin to yield xanthotoxol or the aromatic-carbon hydroxylation of xanthotoxin to form 5-hydroxyxanthotoxin, which are subsequently glycosylated by UGT33 enzymes to form their less-toxic glucosides. In contrast, impaired CYP6AE19–UGT33 coordination in the oligophagous *Helicoverpa assulta* was associated with reduced xanthotoxin detoxification and high sensitivity to this compound. Together, these findings provide direct evidence for coordinated Phase I–Phase II detoxification of a plant defensive compound in insects and show that functional divergence in this pathway contributes to interspecific differences in plant toxin tolerance, which are associated with contrasting dietary breadth in closely related herbivorous insects.

## Abstract 2

The mechanisms by which herbivorous insects coordinate detoxification pathways to counter plant defences and diversify host use remain unclear. This study shows that a cooperative CYP6AE19-UGT33 pathway drives furanocoumarin detoxification in Helicoverpa, highlighting a molecular basis for variation in toxin tolerance and host range.

## Introduction

Plants are not intrinsically ideal food for insects, as they deploy a diverse repertoire of defensive secondary metabolites that are toxic, anti-digestive, or antifeedant [1]. The breadth of an insect’s capacity to detoxify these phytochemicals largely determines its dietary specialisation. Oligophagous species typically harbour detoxification systems optimised for one or a few classes of plant metabolites and therefore exhibit narrow host ranges, whereas polyphagous species possess broader enzymatic repertoires that neutralise disparate compounds and thus exploit a wider spectrum of hosts [2]. This ecological matching underlies the interaction between crops and their pest insects. Accordingly, elucidating the molecular mechanisms by which pests detoxify plant defensive compounds is pivotal for designing strategies that disrupt crop–pest associations and advance sustainable pest management.

Insects have evolved diverse mechanisms to cope with xenobiotic stress, among which biotransformation is one of the most fundamental. This process involves the enzymatic transformation of exogenous compounds into metabolites through a series of catalytic reactions. As a result, many xenobiotics become more hydrophilic and are more readily excreted, thereby reducing their toxicity and contributing to detoxification. Biotransformation is generally mediated by Phase I and Phase II enzymes, which can act independently or in concert depending on the substrate [3]. Phase I reactions—also referred to as primary metabolism or functionalisation reactions—introduce or expose polar functional groups (–OH, –NH_2_, –SH, –COOH, etc.) through oxidation, reduction, or hydrolysis [4,5]. The major enzymes responsible for these transformations include cytochrome P450 monooxygenases (P450s), flavin-containing monooxygenases (FMOs), and esterases, among which P450s play a predominant role [6–10]. Phase II reactions—also referred to as secondary metabolism or conjugation reactions—further modify Phase I products or parent compounds through conjugation with endogenous molecules such as sugars, glutathione, or sulfate groups, generating more water-soluble conjugates [3,5]. Typical conjugation pathways include glycosylation (catalysed by UDP-glycosyltransferases, UGTs), glutathione conjugation (via glutathione S-transferases, GSTs), sulfation (via sulfotransferases), and acetylation (via N-acetyltransferases) [3,10–12]. While UGTs have been less extensively studied than P450s, recent research has increasingly recognised their important roles as Phase II enzymes involved in the detoxification of plant secondary metabolites (PSMs) in insects [13]. Despite these advances, most previous studies have focussed on the role of individual enzymes—for instance, a specific P450 or UGT—in the detoxification of single or limited substrates. Far fewer have explored how Phase I and Phase II reactions operate in a coordinated and integrated manner within insect xenobiotic metabolism. Understanding how these two detoxification phases interact is essential for elucidating the molecular mechanisms underlying insect adaptation to PSMs.

The cotton bollworm, *Helicoverpa armigera* (Hübner), was historically restricted to the Old World—Asia, Europe, Africa, and Australasia—but has recently expanded its range across South and Central America [14,15]. As one of the most destructive agricultural pests worldwide, *H. armigera* imposes enormous economic losses. In the Old World, losses attributable to *H. armigera* alone, including yield reduction and control expenses, have been estimated at approximately US$5 billion. Following its invasion into the New World, damage to Brazilian agriculture caused by *Helicoverpa* species between 2012 and 2014 was estimated at US$0.8–2 billion [14]. A major factor underlying the remarkable destructiveness of *H. armigera* is its extreme polyphagy, with this species feeding on more than 300 host plant species across 68 families [16]. This broad host range is supported by a complex and diverse detoxification enzyme system, along with high transcriptional plasticity in response to different host plants [17]. For instance, our previous work demonstrated that the cytochrome P450 CYP6AE gene cluster enables *H. armigera* to detoxify xanthotoxin from citrus (Rutaceae) and 2-tridecanone from tomato (Solanaceae) [18]. Although functional studies such as those on CYP6AE have established links between detoxification enzymes and specific PSMs, the functions of other detoxification enzymes, particularly those outside the cytochrome P450 superfamily, remain largely unexplored in a systematic manner.

In contrast to the highly polyphagous *H. armigera*, the closely related sibling species, the oriental tobacco budworm *Helicoverpa assulta* (Guenée), is an oligophagous specialist primarily associated with Solanaceae plants, including tobacco, hot pepper and eggplant [19,20]. A recent comparative study showed that *H. assulta* was less tolerant than *H. armigera* to seven of 12 tested PSMs, including coumarin, capsaicin, quercetin, gossypol, rutin, xanthotoxin and flavone, whereas it showed higher tolerance to nicotine, a characteristic defensive compound of tobacco [21]. These contrasting tolerance profiles suggest that differences in the ability to cope with PSMs may have contributed to the divergent dietary breadth of these two closely related species. However, despite the long-recognised difference in host range between *H. armigera* and *H. assulta*, the role of detoxification mechanisms in this divergence remains poorly understood.

Here, we leveraged the *Helicoverpa* system to investigate how detoxification enzymes contribute to adaptation to PSMs and host-range divergence. To this end, we combined comparative genomic analyses, CRISPR/Cas9-mediated knockouts, metabolomic profiling and biochemical assays to characterise the UGT33 family and its coordinated function with the cytochrome P450 enzyme CYP6AE19 in the detoxification of xanthotoxin, a furanocoumarin. Comparative analysis of the polyphagous *H. armigera* and the oligophagous *H. assulta* further revealed that impairment of the CYP6AE19–UGT33 pathway is associated with reduced xanthotoxin detoxification in *H. assulta*. This study therefore addresses three key questions: (i) whether expansion and functional diversification of the UGT33 family contribute to plant toxin detoxification in *Helicoverpa*; (ii) how Phase I cytochrome P450s and Phase II UDP-glycosyltransferases act together to detoxify PSMs; and (iii) whether divergence in this coordinated detoxification pathway helps explain differences in dietary breadth between closely related polyphagous and oligophagous species.

## Results

### Comparative characterisation of the UGT superfamily in *Helicoverpa* and related noctuid species

To comprehensively characterise the UGT superfamily of *H. armigera*, we leveraged four chromosome-level reference genomes derived from geographically distinct strains: Ha-GR (NCBI accession no. GCA_040954515.1) from Australia, Oceania; Ha-SCD (GCA_023701775.1) from Côte d’Ivoire, Africa; Ha-96S (GCA_030705265.1) from China, Asia and Ha-UK (GCA_963930815.1) from United Kingdom, Europe. Using bioinformatic approaches, we identified 49, 62, 47 and 47 UGT genes in the Ha-GR, Ha-SCD, Ha-96S and Ha-UK genomes, respectively (S1–S4 Figs). The major discrepancy among the four genomes occurred on chromosome 28 of the Ha-SCD assembly, where two UGT33 family gene clusters appeared to have been duplicated. Specifically, UGT33 cluster 1 (6.58–6.62 Mb) was duplicated at 8.84–8.89 Mb, and UGT33 cluster 2 (6.81–6.87 Mb) was duplicated at 9.04–9.07 Mb. To determine whether these duplications represented genuine genomic events or assembly artefacts, we compared the sequencing depth of the regions encompassing these loci (6.55–6.88 Mb and 8.82–9.08 Mb) with that of the entire chromosome 28. The average sequencing depths of these two regions (64.79× and 62.49×) were approximately half of the chromosome-wide average (105.40×) (S1 Table). Further resequencing of 10 Ha-SCD individuals revealed a similar pattern (S1 Table). To verify this, we performed genomic qPCR on four representative UGT33 genes: *UGT33J1* and *UGT33B5,* which are single-copy in the Ha-SCD reference genome, and *UGT33B3* and *UGT33M1*, which are shown as duplicated. The relative copy numbers of all four genes in Ha-SCD ranged from 0.83 to 1.07 compared with Ha-96S (S8 Fig), confirming the absence of any genuine copy number increase. Taken together, these results indicate that the apparent duplications of UGT33 clusters in the Ha-SCD genome are most likely assembly artefacts rather than true gene expansions. After correcting for these, the Ha-GR, Ha-SCD, Ha-96S and Ha-UK genomes contain 49, 47, 47 and 47 UGT genes, respectively (Fig 1A).

**Fig 1:**
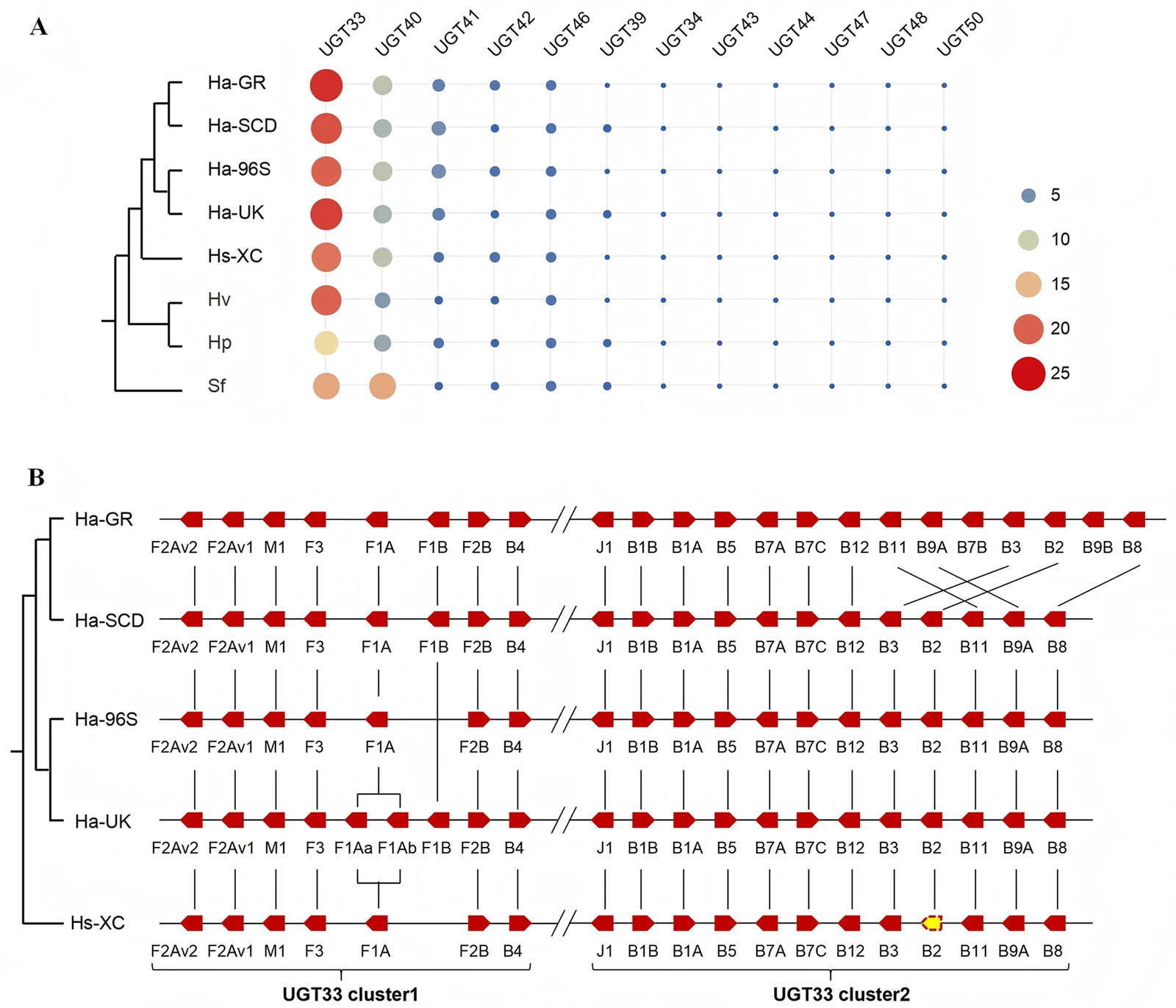
Composition and syntenic analysis of the UGT superfamily in *Helicoverpa*, *Heliothis* and *Spodoptera.* **(A)** Variation in the number of UGT family genes across the genomes of four *H. armigera* strains (Ha-GR from Oceania, Ha-SCD from Africa, Ha-96S from Asia and Ha-UK from Europe), *Helicoverpa assulta* (Hs-XC), *Heliothis viriplaca* (Hv), *Heliothis peltigera* (Hp), and *Spodoptera frugiperda* (Sf). Phylogenetic relationship among species/strains inferred from the shared BUSCO gene sequences (S11 Fig). The colour and size of each circle indicate the number of genes within each UGT family. The panel was visualised using TBtools-II [64]. **(B)** Syntenic analysis of the UGT33 family across four *H. armigera* strains and *H. assulta*. Genes are shown in their correct orientation and order, but physical distances are not to scale. The dashed arrow filled in yellow indicate pseudogene UGT. The data underlying this figure can be found at S1 Data.

We then annotated the complete set of UGT genes in *H. assulta*, a closely related but oligophagous species of *H. armigera*, as well as in two *Heliothis* species phylogenetically close to the genus *Helicoverpa*, namely *Heliothis peltigera* and *Heliothis viriplaca* (Figs 1A and S5–S7). We also compared these UGT genes with the previously published UGT repertoire of *Spodoptera frugiperda*, a more distantly related noctuid species within the family Noctuidae (Fig 1A). Phylogenetic analysis classified these UGTs into 12 families: UGT33, UGT34, UGT39, UGT40, UGT41, UGT42, UGT43, UGT44, UGT46, UGT47, UGT48 and UGT50 (Figs 1A and S1–S7). Among these families, UGT33 and UGT40 showed the most prominent expansions, accounting for 34%–50% and 15%–34% of the total UGT repertoire, respectively. In *Helicoverpa* and *Heliothis*, the UGT33 family was more extensively expanded than the UGT40 family, with UGT33 gene numbers generally more than twice those of UGT40, except in *H. peltigera*, where the difference was closer to 2-fold. In contrast, *S. frugiperda* contained similar numbers of UGT33 and UGT40 genes.

We next performed a detailed analysis of the UGT33 family. dN/dS analysis showed that nearly all UGT33 genes had dN/dS ratios below 1 (S10 Fig), suggesting that this family has generally evolved under purifying selection and that the encoded proteins are likely subject to functional constraint. Notably, the dN/dS ratios of UGT33 genes in *Helicoverpa* were significantly lower than those observed in *Heliothis*, suggesting that the UGT33 family in *Helicoverpa* does not show stronger evidence of accelerated protein sequence evolution.

Comparison between the polyphagous *H. armigera* and its closely related oligophagous relative *H. assulta* revealed only modest differences in UGT gene number. *H. assulta* contained 44 UGT genes, only 6%–10% fewer than the 47–49 genes identified in *H. armigera* (Fig 1A). Likewise, the UGT33 family included 19 genes in *H. assulta*, only 5%–17% fewer than the 20–23 members found in *H. armigera* (Figs 1B and S1–S5). In addition, only one UGT33 gene in *H. assulta*, UGT33B2, was predicted to be a pseudogene (Fig 1B). These results indicate that the contrasting host ranges of *H. armigera* and *H. assulta* are unlikely to be explained simply by differences in UGT or UGT33 gene number. Instead, they may be more closely linked to functional divergence among UGT proteins.

Within *H. armigera*, the UGT33 family comprised 20–23 members, accounting for 43%–47% of the total UGT genes in the four genomes. With the exception of UGT33T1, which is located on a separate chromosome, all other UGT33 genes were organised into two gene clusters on the same chromosome (S1–S4 Figs). Despite the overall conservation of this clustered organisation, several strain-specific differences were observed. *UGT33F1B* was absent from the Ha-96S strain, whereas *UGT33F1A* was duplicated in the Ha-UK strain. In the Ha-GR strain, the arrangement of seven genes from *UGT33B11* to *UGT33B8* differed from that in the Ha-SCD, Ha-96S and Ha-UK strains, and this region contained an additional gene, *UGT33B9B*, that was absent from the other three genomes (Fig 1B). In addition, *UGT33F2Av1* and *UGT33F2Av2* possessed distinct exon 1 sequences but shared exons 2–4, a pattern also observed for *UGT46A3v1* and *UGT46A3v2* (S9 Fig).

We further examined the expression profiles of *H. armigera* UGT33 genes across developmental stages and host plant diets. RNA-seq data from seven developmental stages, including eggs, second-instar larvae, fifth-instar larvae, female pupae, male pupae, female adults and male adults, showed that most UGT33 genes were highly expressed in larval and adult stages but expressed at low levels in eggs and pupae (S12 Fig). This pattern suggests a potential role for the UGT33 family in environmental adaptation during active feeding and reproductive stages. Midgut transcriptome analysis of fifth-instar larvae fed on five different host plants, including large bullwort, kumquat, cotton, maize and tobacco, further showed that 10 UGT33 genes were differentially regulated in response to at least one host plant compared with larvae fed on artificial diet (S12 Fig). These diet-responsive expression patterns suggest that multiple UGT33 genes may be involved in host plant adaptation.

Together, these comparative genomic and transcriptomic analyses identify the UGT33 family as a major and dynamically diversified UGT lineage in *Helicoverpa*. Although most UGT33 genes appear to be maintained under purifying selection, their extensive copy number variation, strain-specific structural rearrangements, alternative exon usage and host plant-responsive expression suggest potential functional diversification. Moreover, the similar sizes of the UGT33 family in the polyphagous *H. armigera* and the oligophagous *H. assulta* indicate that differences in host range are unlikely to be driven simply by gene number. We therefore focussed on the *Helicoverpa* UGT33 family to functionally test whether divergence among these enzymes contributes to the detoxification of PSMs.

### Knockout of the UGT33 gene clusters in *H. armigera* increases sensitivity to furanocoumarin

To investigate the role of the UGT33 family of *H. armigera* in the detoxification of PSMs, we generated gene cluster knockouts of the UGT33 family in the Ha-SCD strain using a dual single-guide RNA (sgRNA)-directed CRISPR-Cas9 system [18]. Two UGT33 gene clusters were targeted: UGT33 cluster 1 (abbreviated as 33c1), containing eight UGT genes (*UGT33F2Av2* to *UGT33B4*), and UGT33 cluster 2 (abbreviated as 33c2), containing 12 genes (*UGT33J1* to *UGT33B8*) (Fig 2A). This resulted in the generation of the SCD-d33c1 strain homozygous for the UGT33 cluster 1 knockout (Figs 2A, S13, and S15), the SCD-d33c2 strain homozygous for the UGT33 cluster 2 knockout (Fig 2B and 2C) and the SCD-d33c1c2 strain homozygous for both the UGT33 cluster 1 and 2 (Figs 2A, S14, and S15). The successful generation of these cluster knockout strains also provides indirect evidence that *UGT33* cluster 1 and cluster 2 in the Ha-SCD strain have not undergone true duplication.

**Fig 2:**
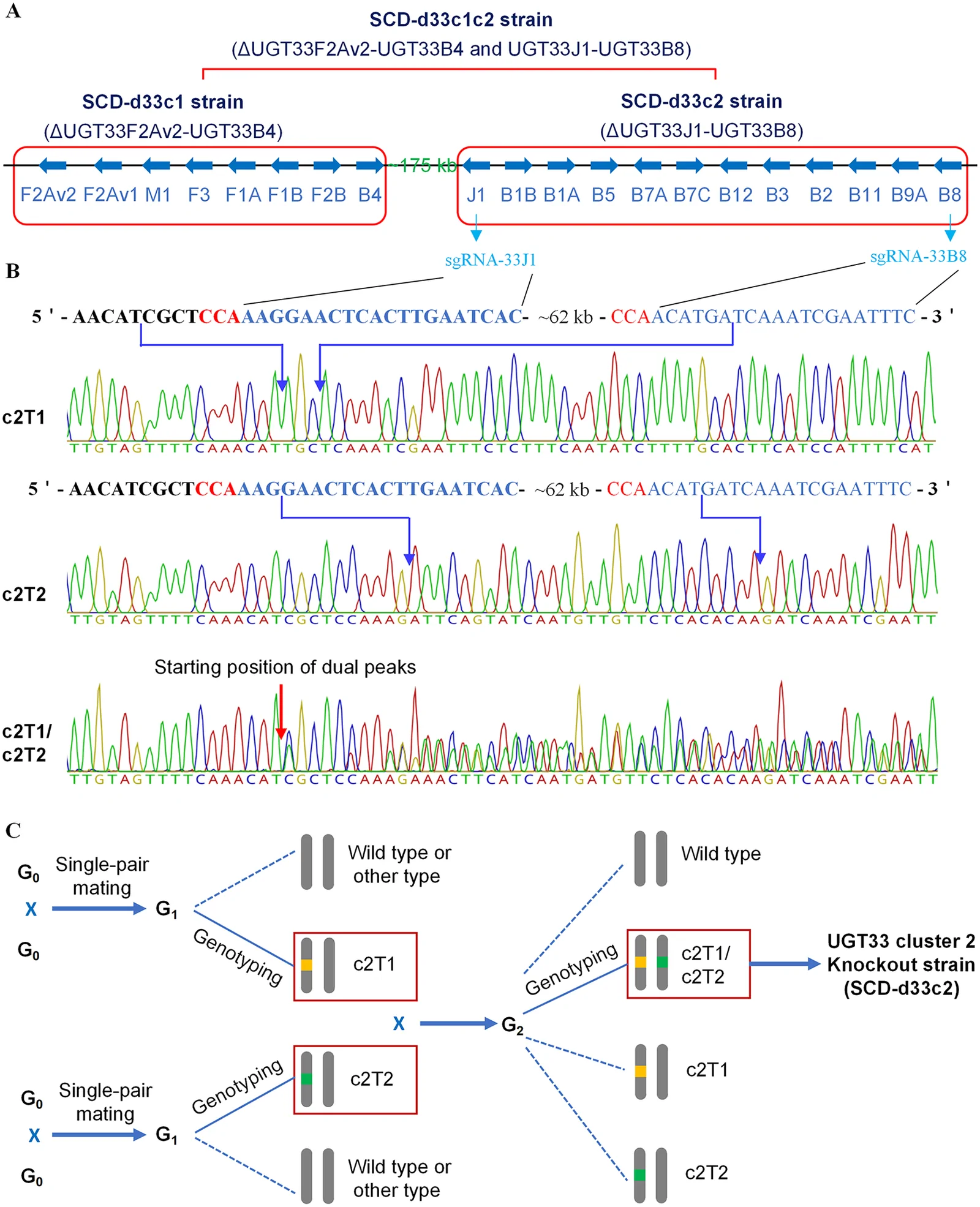
CRISPR-Cas9-mediated individual and combined knockout of two UGT33 gene clusters in *H. armigera.* **(A)** The SCD-d33c1 strain carries a deletion of eight UGT33 genes in cluster 1. The SCD-d33c2 strain carries a deletion of 12 UGT33 genes in cluster 2. The SCD-d33c1c2 strain contains both deletions. The UGT genes are shown in their correct orientation and order. **(B, C)** Representative sequencing chromatograms and schematic diagram illustrating the construction of the SCD-d33c2 strain. The target sequences of the sgRNAs are shown in blue, and the PAM sequences in red. c2T1 and c2T2 represent two distinct genotypes generated by the deletion of an approximately 62 kb genomic fragment between two sgRNAs targeting *UGT33J1* and *UGT33B8*, respectively. These genotypes resulted from imprecise DNA repair through the non-homologous end joining (NHEJ) pathway following CRISPR-Cas9–induced double-strand breaks. c2T1/c2T2 represent overlapping chromatogram peaks of c2T1 and c2T2 within a single individual, indicating the coexistence of both genotypes and confirming a homozygous knockout of the UGT33 cluster 2.

Bioassays were conducted to evaluate changes in the sensitivity of the SCD-d33c1c2 strain relative to the background Ha-SCD strain using eight PSMs representing distinct host-associated chemical classes: gossypol, a Malvaceae-associated sesquiterpene; DIMBOA, a Poaceae benzoxazinoid; nicotine, a Solanaceae alkaloid; flavone, a broadly distributed flavonoid; and four furanocoumarins—xanthotoxin, imperatorin, bergapten and isoimperatorin—typically associated with Apiaceae and Rutaceae. Among these compounds, the SCD-d33c1c2 strain displayed markedly increased sensitivity to the furanocoumarins xanthotoxin and imperatorin, whereas no significant increase in sensitivity was detected for bergapten, isoimperatorin, gossypol, flavone, DIMBOA or nicotine (Fig 3A–3H). These results suggest that the 20 genes within the two UGT33 clusters harbour key determinants of xanthotoxin and imperatorin detoxification in *H. armigera*.

**Fig 3:**
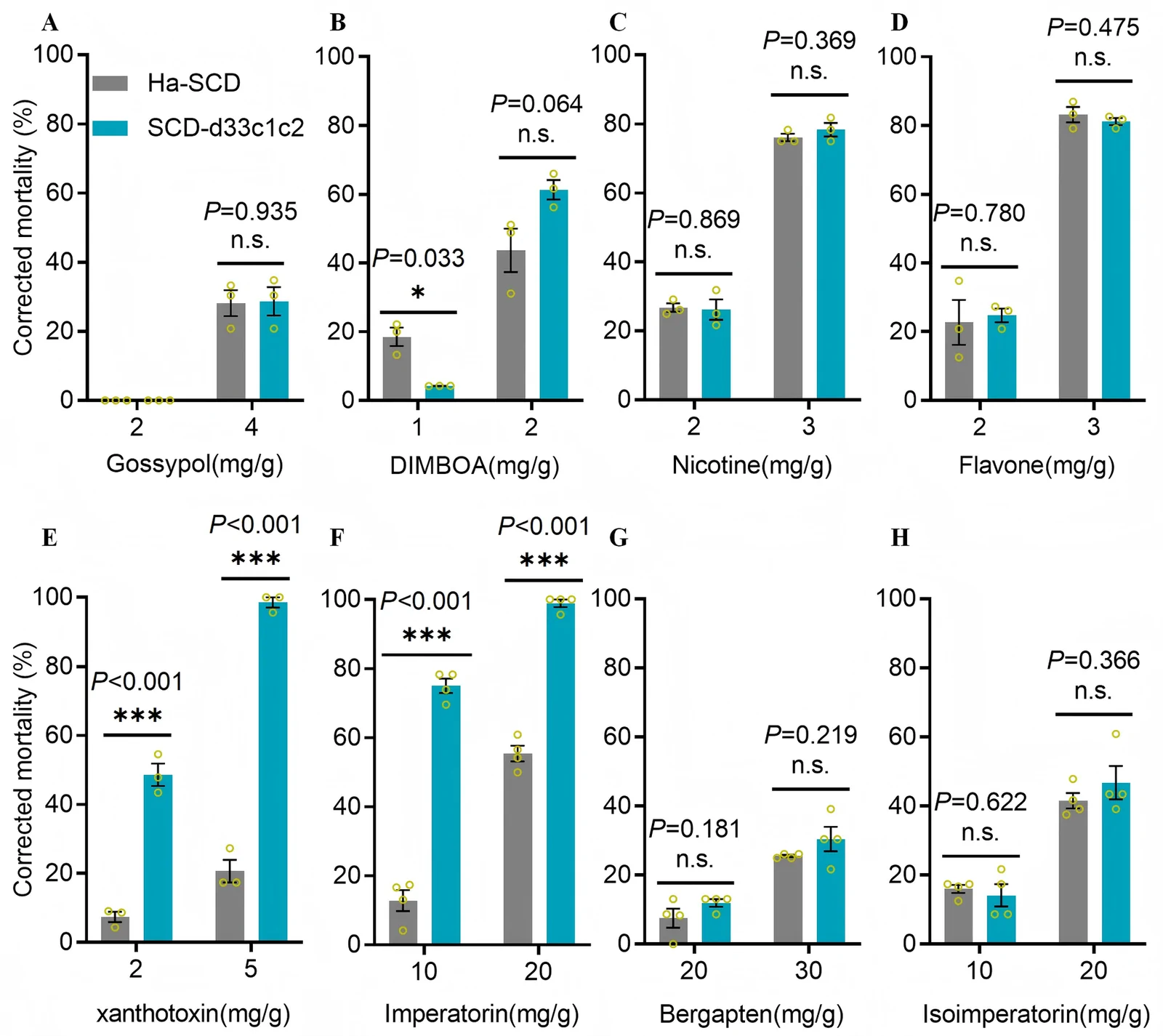
Responses of the SCD-d33c1c2 strain and its parental Ha-SCD strain of *H. armigera* to eight phytotoxins. The SCD-d33c1c2 strain harbours deletions of eight UGT33 genes in cluster 1 and 12 UGT33 genes in cluster 2. Values are means of corrected mortality ± SEM, *n* = 3 or 4 biologically independent samples, 24 larvae were used as a biologically independent sample. Unpaired *t* tests were used for statistical comparisons (*P < 0.05; ***P < 0.001; ns, not significant). The data underlying this figure can be found at S1 Data.

To further pinpoint the specific gene cluster involved, bioassays were performed using the single-cluster knockout strains SCD-d33c1 and SCD-d33c2 to assess their sensitivity to four furanocoumarin PSMs. Relative to the background strain, SCD-d33c2 showed a pronounced increase in sensitivity to xanthotoxin and imperatorin, but little or no change in response to bergapten or isoimperatorin (Fig 4A–4D). By comparison, the SCD-d33c1 strain exhibited no significant increase, or even a slight decrease, in sensitivity to any of the four compounds (Fig 4A–4D). Collectively, these data identify UGT33 cluster 2—comprising 12 genes—as the principal contributor to xanthotoxin and imperatorin detoxification in *H. armigera*.

**Fig 4:**
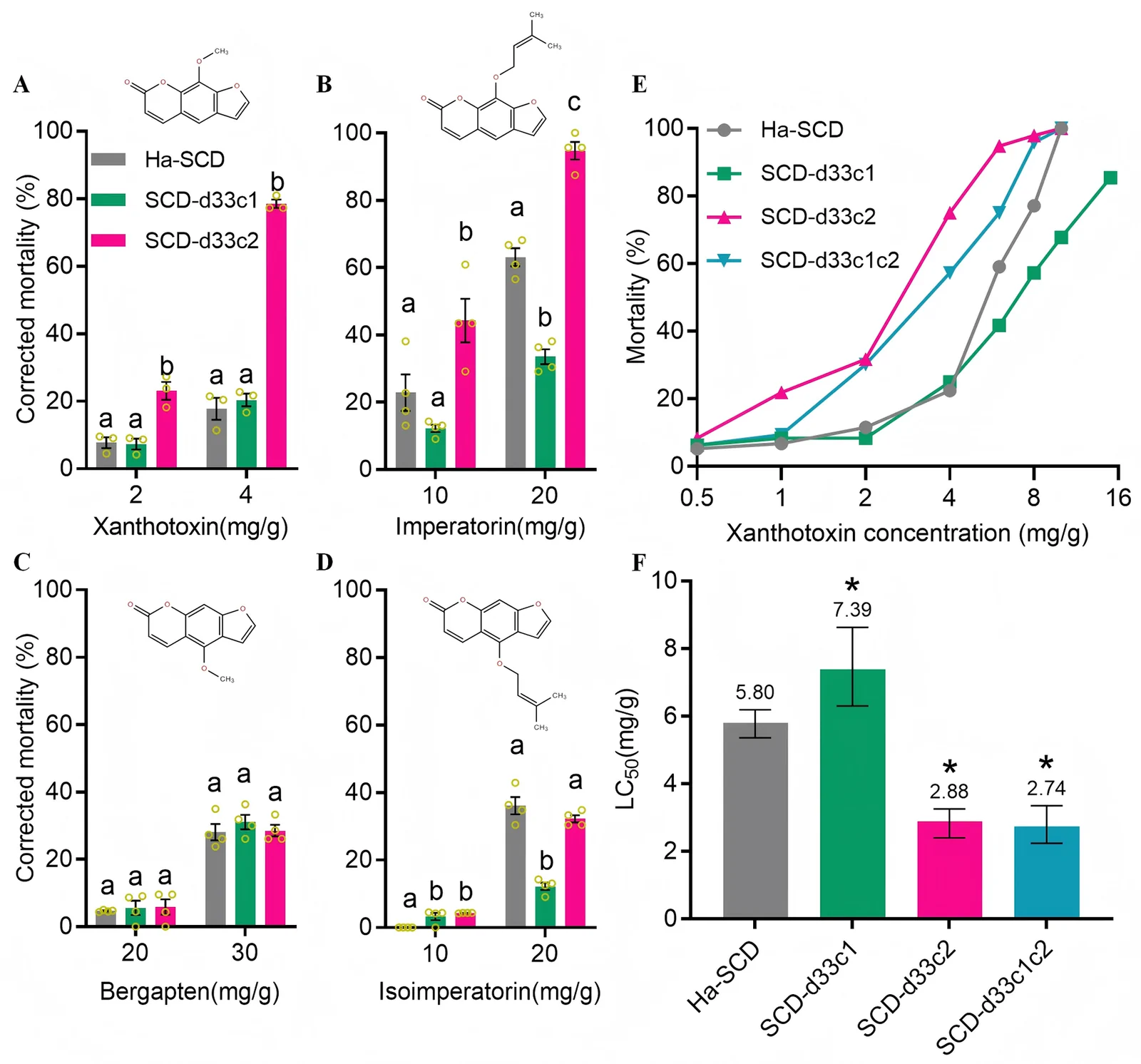
Responses of *H. armigera* UGT33 cluster knockout strains and their parental Ha-SCD strain to furanocoumarin phytotoxins. The SCD-d33c1 and SCD-d33c2 strains were generated from Ha-SCD by knocking out UGT33 clusters 1 and 2, respectively, while the SCD-d33c1c2 strain harbours deletions in both clusters. **(A–D)** Responses of *H. armigera* larvae from Ha-SCD, SCD-d33c1 and SCD-d33c2 strains to four furanocoumarin phytotoxins. Molecular structures of four compounds are shown. Values are means ± SEM, *n* = 3 or 4 biologically independent samples, 24 larvae were used as a biologically independent sample. Significant differences (*P* < 0.05) are denoted using different letters above bars as determined by one-way ANOVA with Tukey HSD. **(E, F)** Dose–response curves and LC_50_ values of larvae from the Ha-SCD, SCD-d33c1, SCD-d33c2, and SCD-d33c1c2 strains exposed to xanthotoxin. Asterisks indicate that the 95% fiducial limits of LC_50_ values between knockout and parental Ha-SCD strains do not overlap. The data underlying this figure can be found at S1 Data.

Furthermore, to provide a more quantitative assessment of detoxification ability, the median lethal concentration (LC_50_) was determined to evaluate the effect of UGT33 cluster knockout on xanthotoxin toxicity. The LC_50_ assays revealed a 2.02-fold increase in sensitivity in the SCD-d33c2 strain compared with the Ha-SCD strain, supported by non-overlapping 95% fiducial limits of LC_50_ values between the two strains (Fig 4E and 4F; S2 Table). The level of increased sensitivity in SCD-d33c2 was comparable to that observed in the SCD-d33c1c2 strain (2.12-fold) relative to the Ha-SCD strain (Fig 4E and 4F; S2 Table). Taken together, these findings confirm that one or more genes within UGT33 cluster 2 are responsible for the detoxification of the furanocoumarin xanthotoxin in *H. armigera*.

### Coordinated action of cytochrome P450 and UGTs enhances detoxification of xanthotoxin in *H. armigera*

Because UGTs typically conjugate sugar moieties to nucleophilic groups such as hydroxyl, carboxyl, sulfhydryl, amino, or substituted amines [22], and xanthotoxin lacks such functional groups, we hypothesised that xanthotoxin might first be converted into glycosylatable intermediates through the catalytic activity of Phase I enzymes such as cytochrome P450s, before being further glycosylated by UGTs. To test this hypothesis, we performed untargeted metabolomic analyses of *H. armigera* faeces after feeding on xanthotoxin. Compared with the faeces from larvae fed on a control diet, those from xanthotoxin-fed larvae showed a marked enrichment of potential xanthotoxin metabolites—xanthotoxol and 5-hydroxyxanthotoxin—as well as their corresponding glycosylated derivatives (S16A Fig). Targeted metabolomic analyses using UPLC-MS/MS further confirmed the presence of these metabolites (Figs 5B and S16B).

**Fig 5:**
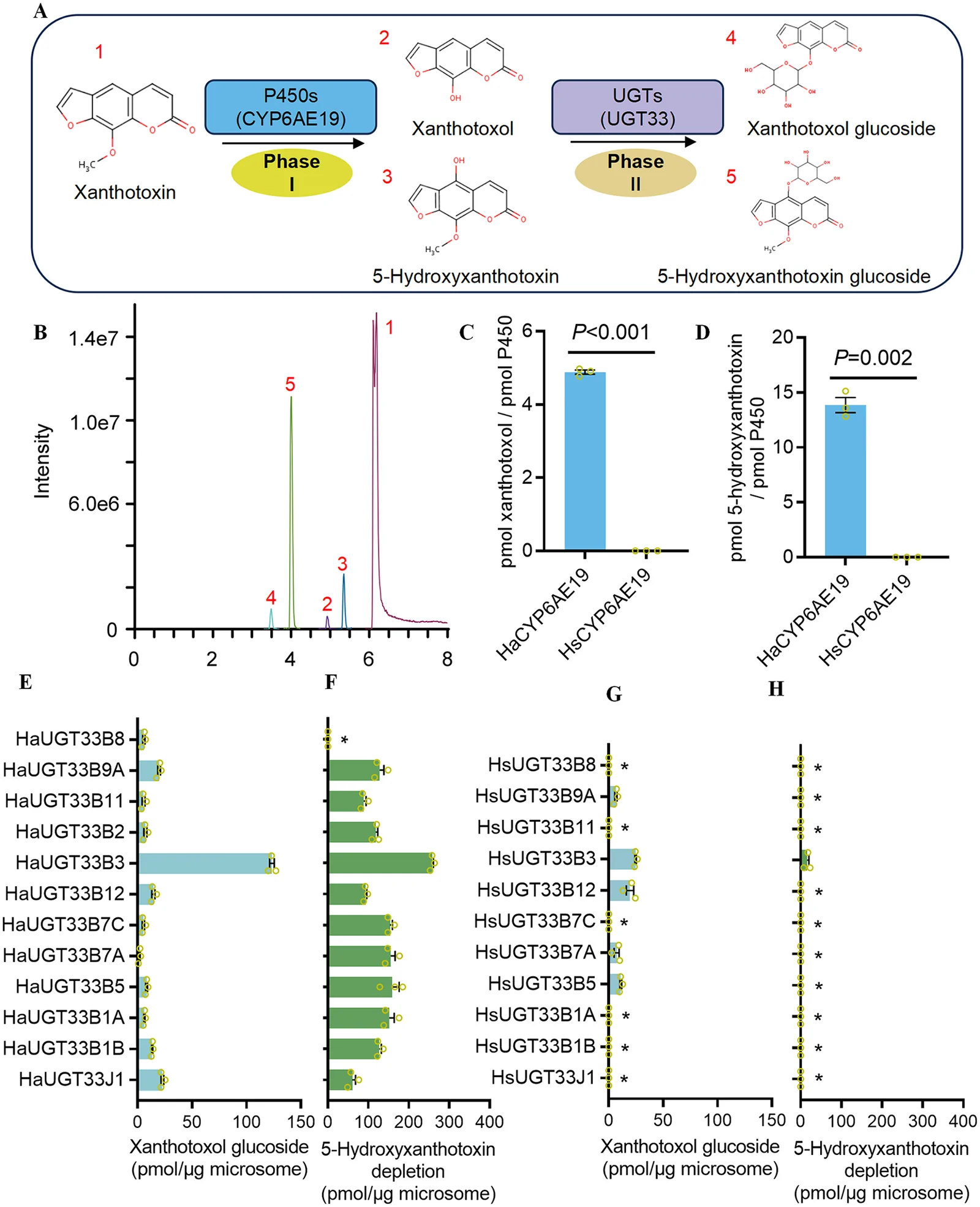
Sequential metabolism of xanthotoxin by CYP6AE19 and UGT33 enzymes in *Helicoverpa.* **(A)** Proposed metabolic pathway of xanthotoxin in *H. armigera*. The Phase I cytochrome P450 CYP6AE19 catalyses either the O-dealkylation of xanthotoxin to yield xanthotoxol or the aromatic-carbon hydroxylation of xanthotoxin to form 5-hydroxyxanthotoxin, which are subsequently glycosylated by Phase II UGT33 enzymes to form their respective glucosides. **(B)** Metabolites in larval faeces after feeding on xanthotoxin-supplemented diet. 1, xanthotoxin; 2, xanthotoxol; 3, 5-hydroxyxanthotoxin; 4, xanthotoxol glucoside; 5, 5-hydroxyxanthotoxin glucoside. Low-abundance MRM traces were scaled for visualisation as indicated (xanthotoxol, ×20; 5-hydroxyxanthotoxin, ×10; xanthotoxol glucoside, ×10). **(C, D)** In vitro metabolism of xanthotoxin by recombinant CYP6AE19 protein. Formation of the xanthotoxin-derived metabolites xanthotoxol (C) and 5-hydroxyxanthotoxin (D) was quantified after incubation with *H. armigera* CYP6AE19 (HaCYP6AE19) and *H. assulta* CYP6AE19 (HsCYP6AE19). **(E, F)** In vitro metabolism of xanthotoxol (E) and 5-hydroxyxanthotoxin (F) by recombinant *H. armigera* UGT33 proteins. **(G, H)** In vitro metabolism of xanthotoxol (G) and 5-hydroxyxanthotoxin (H) by recombinant *H. assulta* UGT33 proteins. **(C–H)** Error bars represent mean values ± SEM (*n* = 3). **(E–H)** Asterisks indicate that no significant metabolism was detected. The data underlying this figure can be found at S1 Data.

Our previous study demonstrated, using substrate depletion assays, that the cytochrome P450 enzyme CYP6AE19 is capable of metabolising xanthotoxin, although the metabolic products remained unidentified [18]. In the present study, UPLC-MS/MS analysis of the CYP6AE19-mediated reaction products revealed the presence of xanthotoxol and 5-hydroxyxanthotoxin (Fig 5C and 5D), providing preliminary evidence that P450-mediated metabolism of xanthotoxin generates glycosylatable intermediates. To further investigate the subsequent glycosylation step, 12 UGT proteins encoded by genes within UGT33 cluster 2 of *H. armigera* were functionally expressed in a lepidopteran cell line using a baculovirus expression system. Western blot analysis confirmed successful expression of all 12 recombinant UGT33 proteins at the expected molecular sizes (S17A and S17B Fig). All recombinant UGT33 proteins showed variable activity towards the model substrate 1-naphthol (1-NA) (S18A Fig). The formation of xanthotoxol glucoside and the depletion of 5-hydroxyxanthotoxin were used as the criteria  to assess the metabolic activity of each UGT against xanthotoxol and 5-hydroxyxanthotoxin respectively. Among the 12 UGT33 proteins assayed, all 12 exhibited varying capacities to glycosylate xanthotoxol to xanthotoxol glucoside, and 11 showed detectable metabolic activity towards 5-hydroxyxanthotoxin to different extents (Fig 5E and 5F). Notably, recombinant UGT33B3 protein showed the highest catalytic activity towards both xanthotoxin-derived intermediates (Fig 5E and 5F). Taken together, these results demonstrate that xanthotoxin is first metabolised by CYP6AE19 to produce xanthotoxol and 5-hydroxyxanthotoxin, which are subsequently glycosylated by multiple UGT33 enzymes to form their corresponding glucoside conjugates in *H. armigera* (Fig 5A).

To further confirm in vivo that xanthotoxin detoxification requires the coordinated actions of cytochrome P450 CYP6AE19 and UGT33 enzymes, two knockout strains were generated using molecular genotyping and CRISPR–Cas9 technologies: the SCD-d6AE-d33c2 strain, in which nine CYP6AE genes and 12 UGT33 genes were deleted simultaneously, and the SCD-d6AE19-d33c2 strain, in which *CYP6AE19* and the 12 UGT33 genes of cluster 2 were removed (Figs 6, S20, and S21). To assess whether the CYP6AE cluster and UGT33 cluster 2 act independently or synergistically in xanthotoxin detoxification, we compared the LC_50_ values of single- and double-knockout *H. armigera* strains. The Ha-SCD strain exhibited an LC_50_ of 5.80 mg/g (95% FL: 5.36–6.19). Knockout of the CYP6AE cluster (SCD-d6AE) or UGT33 cluster 2 (SCD-d33c2) significantly decreased the LC_50_ to 1.39 mg/g (95% FL: 1.14–1.70) and 2.88 mg/g (95% FL: 2.40–3.26), respectively (Fig 6; S2 Table). Based on the independence model, the expected LC_50_ for the double knockout (SCD-d6AE-d33c2) under independent action was 0.69 mg/g [(LC_50_ of SCD-d6AE) × (LC_50_ of SCD-d33c2)/(LC_50_ of Ha-SCD)]. However, the observed LC_50_ (1.18 mg/g, 95% FL: 0.95–1.44) was substantially higher, indicating a less-than-additive effect. Furthermore, the overlapping 95% fiducial limits between SCD-d6AE and both SCD-d6AE-d33c2 and SCD-d6AE19-d33c2 suggest that the combined disruption did not further increase xanthotoxin sensitivity (Fig 6; S2 Table). Collectively, these results indicate that CYP6AE19 and UGT33 cluster 2 function in a coordinated yet non-synergistic manner in mediating xanthotoxin detoxification.

**Fig 6:**
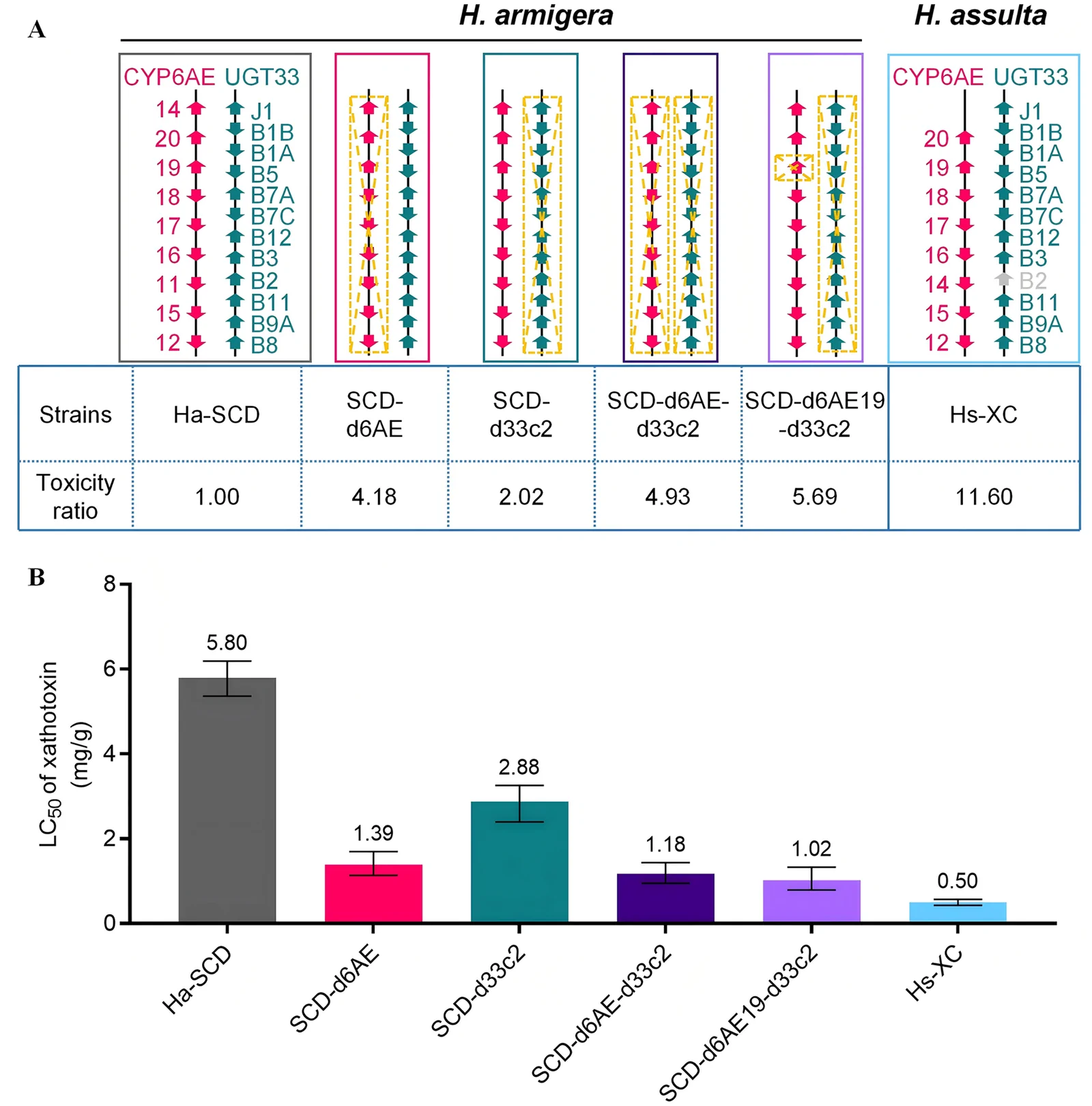
Coordinated action of CYP6AE and UGT33 enzymes enhances xanthotoxin detoxification in *H. armigera.* **(A)** Schematic of *H. armigera* knockout strains and *H. assulta* Hs-XC strain. SCD-d6AE, deletion of a cluster of nine *CYP6AE* genes; SCD-d33c2, deletion of 12 *UGT33* genes in cluster 2; SCD-d6AE-d33c2, double knockout of both clusters; SCD-d6AE19-d33c2: knockout of *CYP6AE19* and the 12 *UGT33* genes in cluster 2. Toxicity ratio was calculated as the LC_50_ of Ha-SCD divided by that of each knockout strain or the *H. assulta* Hs-XC strain. **(B)** Responses of the Ha-SCD, SCD-d6AE, SCD-d33c2, SCD-d6AE-d33c2, SCD-d6AE19-d33c2 and Hs-XC strains to xanthotoxin. LC_50_ values were considered significantly different if their 95% fiducial limits did not overlap. The data underlying this figure can be found at S1 Data.

### Impaired P450–UGT coordination is associated with high xanthotoxin sensitivity in *H. assulta*

To investigate whether species differences in the coordinated P450–UGT pathway are associated with xanthotoxin tolerance, sensitivity to xanthotoxin was compared between the polyphagous *H. armigera* and its closely related oligophagous *H. assulta*. The LC_50_ of *H. assulta* was 0.50 mg/g, which was 11.6-fold lower than that of *H. armigera*, indicating markedly higher sensitivity to xanthotoxin in *H. assulta* (Fig 6).

To examine whether this increased sensitivity is associated with impaired Phase I metabolism, the *H. assulta* CYP6AE19 protein (HsCYP6AE19) was functionally characterised. Recombinant HsCYP6AE19 was successfully expressed using a baculovirus-mediated insect cell expression system, as confirmed by its reduced CO-difference spectrum (S19 Fig). However, in vitro metabolism assays showed that HsCYP6AE19 was unable to metabolise xanthotoxin, and neither xanthotoxol nor 5-hydroxyxanthotoxin was detected as a reaction product (Fig 5C and 5D). This contrasts with *H. armigera* CYP6AE19, which catalyses the conversion of xanthotoxin into these two glycosylatable intermediates.

To examine whether this increased sensitivity was associated with impaired Phase II metabolism, UGT33 proteins encoded by the second UGT33 gene cluster of *H. assulta* were further expressed. Successful expression of recombinant UGT33 proteins was confirmed by western blotting (S17C and S17D Fig) and their enzymatic activity to model substrate 1-NA. Although all recombinant HsUGT33 proteins displayed detectable activity towards 1-NA (S18B Fig), their activity towards xanthotoxin-derived intermediates was limited (Fig 5G and 5H). Among the HsUGT33 proteins tested, only five showed detectable but weak activity in converting xanthotoxol to xanthotoxol glucoside, with the activity of HsUGT33B3 towards xanthotoxol being 5.0-fold lower than that of HaUGT33B3 (Fig 5G). For 5-hydroxyxanthotoxin, detectable metabolism was observed only for HsUGT33B3, with an activity 15.9-fold lower than that of HaUGT33B3 (Fig 5H). Together, these results indicate that HsUGT33 enzymes have substantially reduced metabolic capacity towards xanthotoxin-derived intermediates compared with their *H. armigera* counterparts.

To examine whether the contrasting xanthotoxin detoxification capacities of the two species could be explained by transcriptional induction, midgut transcriptomes were analysed from *H. armigera* and *H. assulta* larvae after 48 h of feeding on a xanthotoxin-containing artificial diet. *CYP6AE19* and three *UGT33* genes were significantly upregulated by xanthotoxin in both species, while three additional *UGT33* genes were specifically induced in *H. armigera* and another three in *H. assulta* (S27 and S28 Fig). Notably, *CYP6AE19* was strongly induced in both species, despite the lack of detectable xanthotoxin-metabolising activity of HsCYP6AE19 in vitro. These results suggest that the reduced xanthotoxin detoxification capacity of *H. assulta* is unlikely to result simply from weaker transcriptional induction of *CYP6AE19*, but is instead associated with reduced catalytic capacity at the protein level.

In conclusion, these results reveal a marked difference in the P450–UGT detoxification cascade between *H. armigera* and *H. assulta*. In *H. armigera*, HaCYP6AE19 catalyses the Phase I conversion of xanthotoxin into xanthotoxol and 5-hydroxyxanthotoxin, which are subsequently glucosylated by HaUGT33 enzymes to form xanthotoxol glucoside and 5-hydroxyxanthotoxin glucoside (Fig 7). In contrast, *H. assulta* HsCYP6AE19 lacks detectable activity towards xanthotoxin, while HsUGT33 enzymes retain only weak activity towards xanthotoxol and 5-hydroxyxanthotoxin (Fig 7). These findings suggest that attenuation of the coordinated P450–UGT detoxification pathway contributes to the reduced ability of *H. assulta* to metabolise xanthotoxin and explain its higher sensitivity to this plant defensive compound.

**Fig 7:**
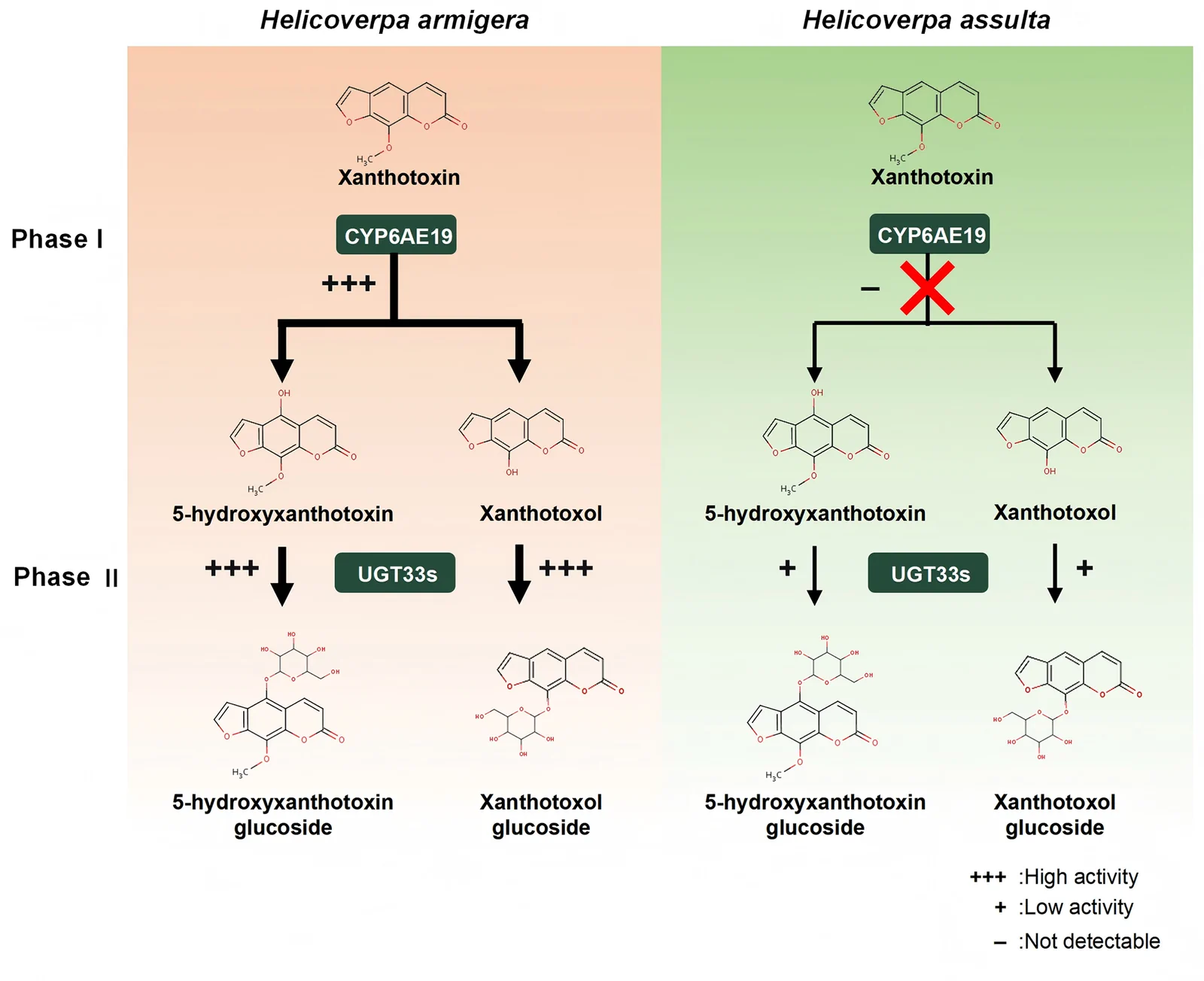
Comparative schematic of the CYP6AE19–UGT33 detoxification cascade mediating xanthotoxin metabolism in *H. armigera* and *H. assulta.* In *H. armigera*, dietary xanthotoxin is efficiently metabolised through a sequential Phase I–Phase II pathway. CYP6AE19 first catalyses O-dealkylation to produce xanthotoxol or aromatic-ring hydroxylation to generate 5-hydroxyxanthotoxin. These hydroxyl-containing intermediates are then glucosylated by UGT33 enzymes to form xanthotoxol glucoside and 5-hydroxyxanthotoxin glucoside, thereby increasing their hydrophilicity and promoting detoxification. In *H. assulta*, HsCYP6AE19 lacks detectable activity towards xanthotoxin, and only a subset of HsUGT33 enzymes show weak glucosylation activity towards xanthotoxol and 5-hydroxyxanthotoxin, indicating attenuation of this coordinated P450–UGT detoxification cascade.

## Discussion

Insects have evolved sophisticated metabolic systems to overcome plant chemical defences, and utilise them as hosts. In this study, we demonstrate that the phase II enzyme UGT superfamily in *Helicoverpa* has undergone a pronounced lineage-specific expansion, with the UGT33 family forming its most dominant clade. Functional analyses in *H. armigera* demonstrated that UGT33 enzymes play a key role in the detoxification of specific furanocoumarins and operate in concert with the Phase I cytochrome P450 enzyme CYP6AE19 during xanthotoxin metabolism. This coordinated pathway converts xanthotoxin into glycosylatable intermediates and subsequently conjugates them into glucosides, establishing a sequential Phase I–Phase II detoxification route in a polyphagous lepidopteran herbivore. By contrast, the corresponding pathway is attenuated in the oligophagous *H. assulta*, where impaired CYP6AE19 activity and reduced UGT33-mediated glycosylation are associated with high xanthotoxin sensitivity. These findings identify P450–UGT metabolic coordination as a key mechanism shaping furanocoumarin detoxification in *Helicoverpa* and illustrate how divergence in detoxification pathways may influence plant toxin tolerance in closely related species.

Our study revealed that the UGT33 family in *H. armigera* has undergone remarkable expansion, accounting for nearly half of all UGT genes identified in this species. Such a major expansion suggests a potential role in environmental adaptation. Previously, our comparative evolutionary and synteny analyses across five *Spodoptera* species demonstrated that the UGT33 family exhibits dynamic lineage-specific changes within this genus [13]. Notably, the oligophagous species *Spodoptera picta* has undergone extensive pseudogenisation of UGT33 genes compared with the other four polyphagous *Spodoptera* species [13]. In contrast, the present study represents the first comprehensive phylogenetic and syntenic analysis of UGT genes among different populations of the same species. Among the 23 UGT33 genes identified in *H. armigera*, 14 genes retained conserved positions and orientations across four geographically distinct populations (originating from Oceania, Africa, Asia and Europe), whereas the remaining nine genes displayed gene gain, loss, or rearrangement. These patterns indicate that the UGT33 family has continued to evolve dynamically even after species divergence, possibly as a response to environmental pressures. This population-specific diversification was particularly evident in the Oceania-derived Ha-GR strain. In Ha-GR, the arrangement of seven genes from *UGT33B11* to *UGT33B8* differed from that in the other three *H. armigera* strains. This pattern may reflect the distinctive geographic and ecological context of Oceania, where physical separation from Afro-Eurasian populations, regional climatic conditions and local agricultural practices may have shaped population-specific evolutionary trajectories. This interpretation is consistent with previous population genomic evidence distinguishing Australasian *H.* armigera conferta from the “Rest of World” *H. armigera armigera* lineage, including genetic differentiation between Australian populations and those from Africa and Asia [23]. Although broader sampling is required, the distinctive UGT33 organisation in Ha-GR suggests that regional evolutionary history may contribute to diversification of detoxification gene families within *H. armigera*. In addition, the observation that different populations of *H. armigera* possess distinct numbers of UGT genes further suggests that the copy number of detoxification genes in insects is not static but evolutionarily dynamic. This variability highlights the importance of conducting pan-genome investigations, which enable a more comprehensive and integrative understanding of the evolutionary diversification and functional adaptation of detoxification enzyme gene families in insects [24].

Using CRISPR/Cas9-mediated knockout combined with bioassays, we demonstrated that the second UGT33 gene cluster plays a critical role in the detoxification of the furanocoumarins xanthotoxin and imperatorin. Notably, knockout of this cluster significantly increased larval sensitivity to xanthotoxin and imperatorin, but not to bergapten or isoimperatorin, which are structurally related compounds within the same furanocoumarin class. Structurally, both xanthotoxin and imperatorin are substituted at position 8 of the psoralen (7H-Furo[3,2-g]chromen-7-one) backbone, with methoxy and prenyloxy groups, respectively. In contrast, bergapten and isoimperatorin carry the same substituents at position 5 of the psoralen core [25]. These positional differences may underlie the substrate specificity of the UGT33 family toward particular furanocoumarins. Alternatively, these structural differences may also affect the preceding Phase I metabolism by cytochrome P450s, thereby limiting the formation of glycosylatable intermediates required for subsequent UGT-mediated conjugation. Interestingly, the SCD-d33c1 strain showed a modest decrease in sensitivity to xanthotoxin compared with the parental Ha-SCD strain. Although this effect was small, it might reflect compensatory regulation between the two UGT33 clusters. One possible explanation is that deletion of UGT33 cluster 1 could induce compensatory upregulation of genes in UGT33 cluster 2, several of which mediate the glycosylation of the xanthotoxin metabolites xanthotoxol and 5-hydroxyxanthotoxin. Such a response could increase phase II conjugation capacity and thereby contribute to the slightly enhanced xanthotoxin tolerance in the SCD-d33c1 strain. This hypothesis could be tested by comparing the expression of UGT33 cluster 2 genes between the SCD-d33c1 and parental Ha-SCD strains after feeding on a xanthotoxin-containing diet. More broadly, although UGT33 cluster 1 also contains several UGT genes, no clear role for this cluster in the detoxification of the PSMs tested here was identified. This does not necessarily indicate that cluster 1 is functionally irrelevant. Its contribution may be masked by functional redundancy with other detoxification enzymes, including other UGTs or enzyme families with overlapping substrate spectra. Alternatively, UGT33 cluster 1 may be specialised for phytochemicals not included in this study. Given the broad host range of *H. armigera* and the diversity of chemical defences encountered in nature, broader phytochemical screening will be needed to define the ecological role of UGT33 cluster 1. Furthermore, simultaneous knockout of both UGT33 gene clusters did not significantly affect susceptibility to DIMBOA, a major defensive benzoxazinoid compound in maize. This finding indicates that the UGT33 clusters in *H. armigera* are not involved in DIMBOA detoxification, and that the previously reported *SfUGT33F32* and its orthologues play a *Spodoptera*-specific role that does not extend to *Helicoverpa* [13]. Collectively, these findings uncover species- and compound-specific functional diversification of the UGT33 family in lepidopteran pests, providing new insights into how gene family expansion contributes to the adaptive evolution of xenobiotic detoxification.

Xanthotoxin, also known as 8-methoxypsoralen, is a representative natural linear furanocoumarin that occurs widely in plants of the Rutaceae and Umbelliferae families. It serves as an important chemical defence compound against herbivorous insects [25–27]. As a classical model compound for studying insect adaptation to host plant defences, xanthotoxin has been extensively investigated, with most studies focussing on its detoxification by cytochrome P450 monooxygenases. Early research, particularly on adapted specialists such as *Papilio polyxenes* and less adapted generalists within the genus *Papilio*, demonstrated that P450 enzymes of the CYP6B subfamily catalyse the metabolism of xanthotoxin and other furanocoumarins. This capacity has been recognised as a key evolutionary innovation underpinning host specialisation in *Papilio* [28–32]. Subsequent work extended these findings to polyphagous lepidopteran pests, showing that CYP6B8 in *Helicoverpa zea*, CYP6AB11 in *Amyelois transitella*, and CYP6AE19 in *H. armigera* can also metabolise xanthotoxin, albeit with lower catalytic efficiency than CYP6B1 in *P. polyxenes* [18,33,34]. More recently, several carboxylesterases, GSTs, and UGTs have been implicated in xanthotoxin detoxification in *Spodoptera litura* [35–37]. However, no prior study has experimentally demonstrated the combined detoxification of xanthotoxin by different classes of detoxification enzymes in insects. Here, we show that xanthotoxin undergoes sequential detoxification via the cooperative actions of the P450 CYP6AE19 and UGT33 enzymes in *H. armigera*, providing an integrated view of multi-enzyme metabolic coordination in insect xenobiotic detoxification.

Notably, this study reveals that a suite of detoxification enzymes in *H. armigera*—including CYP6AE19 and multiple UGT33s—possess the capacity to metabolise xanthotoxin or its downstream intermediates, xanthotoxol and 5-hydroxyxanthotoxin. From an evolutionary perspective, the expansion of the UGT33 family genes provides a genetic reservoir for the metabolism of xanthotoxin and thereby confers potential functional redundancy. Such redundancy prevents the loss of detoxification capacity that might otherwise result from mutation, down-regulation, or inhibition of a single gene, ultimately enhancing the insect’s buffering capacity and adaptive potential when confronted with diverse and fluctuating arrays of PSMs. Relevant to this, *H. armigera* is a typical polyphagous agricultural pest that encounters a wide spectrum of phytochemicals, of which furanocoumarins represent only one class [16,18]. The dramatic expansion of the UGT33 family is therefore unlikely to have arisen specifically in response to xanthotoxin. Rather, it may reflect enzyme promiscuity, whereby a single enzyme can act on multiple substrates [38]. More specifically, UGT33 enzymes may be retained under historical selection pressures imposed by other plant secondary compounds, yet their intrinsic promiscuity allows them, serendipitously, to metabolise xanthotoxin or its intermediates. The combination of enzyme promiscuity and detoxification gene family expansion frequently results in a scenario where one detoxification enzyme can metabolise multiple plant toxins, or multiple enzymes can detoxify a single toxin [18,33,39,40]. Such properties are common among polyphagous insects [18,33,39] and are key determinants enabling their utilisation of a broad range of host plants.

Although the concept of Phase I and Phase II metabolic coupling is well established in biotransformation theory, direct experimental evidence in insects remains scarce. In contrast, mammalian metabolism provides several well-characterised examples. For instance, the environmental toxicant 2,3,7-trichlorodibenzo-p-dioxin (2,3,7-triCDD) is first hydroxylated by CYP1A1 to form 8-hydroxy-2,3,7-triCDD, followed by UGT-mediated glucuronidation to produce its glucuronide conjugates [41]. Similarly, the analgesic codeine is initially O-demethylated by CYP2D6 to generate morphine, which is subsequently glucuronidated by UGTs to form morphine-3- and morphine-6-glucuronides [42]. Such sequential P450–UGT pathways are known to facilitate efficient detoxification and excretion of xenobiotics. In our study, we revealed a comparable mechanism in insects: xanthotoxin is first hydroxylated by CYP6AE19, generating xanthotoxol or 5-hydroxyxanthotoxin, which are then glycosylated by UGT33 enzymes to produce xanthotoxol glucoside and 5-hydroxyxanthotoxin glucoside, respectively. This finding provides compelling systematic evidence of a coordinated Phase I–Phase II biotransformation pathway mediating the detoxification of a plant secondary metabolite in insects, filling an important gap in our understanding of insect detoxification mechanisms. Beyond detoxification, such P450–UGT cooperation may represent a general metabolic strategy in insects. Indeed, previous studies uncovered a P450–UGT coupled biosynthetic pathway in the Burnet moth *Zygaena filipendulae*, where CYP405A2 and CYP332A3 sequentially convert L-isoleucine into cyanohydrin intermediates, which are subsequently glycosylated by UGT33A1 to produce the cyanogenic glucoside lotaustralin, completing the P450–UGT mediated biosynthesis of an insect defensive compound [43]. Together, these findings suggest that cooperative interactions between Phase I and Phase II enzymes may represent a widespread and evolutionarily conserved mechanism of biotransformation, warranting further investigation across diverse taxa and chemical contexts.

Although detoxification enzymes have long been implicated in insect host adaptation, well-resolved examples directly linking specific detoxification mechanisms to host-range divergence are still relatively scarce. Several well-studied examples involve Phase I cytochrome P450 enzymes. In *Papilio* butterflies, functional diversification of furanocoumarin-metabolising CYP6B enzymes is linked to host-range divergence, with generalist species showing broader but less efficient metabolism and specialist species evolving more efficient and substrate-specialised CYP6B activity towards furanocoumarins [44]. In tobacco-adapted races of the polyphagous aphid *Myzus persicae* (*M. persicae* nicotianae), amplification and constitutive overexpression of *CYP6CY3* confer efficient nicotine detoxification and facilitate the host shift to tobacco, demonstrating that P450 evolution can drive host-associated adaptation and differentiation [45]. Phase II enzymes can also shape host-range divergence, as shown by *UGT33F32*-mediated DIMBOA detoxification in *Spodoptera*, which contributes to differences in host plant range between generalist and specialist species [13]. Extending these studies, we identified a sequential CYP6AE19–UGT33 pathway that efficiently detoxifies xanthotoxin in the polyphagous *H. armigera*, whereas the corresponding enzymes in the oligophagous *H. assulta* exhibit substantially lower activity. Consistent with these biochemical differences, *H. assulta* shows markedly greater sensitivity to xanthotoxin in vivo. Xanthotoxin and related furanocoumarins are primarily associated with Apiaceae and Rutaceae, plant families included within the broad host range of *H. armigera* but outside the typical Solanaceae-associated host range of *H. assulta* [19–21]. The concordance among ecological exposure, whole-organism sensitivity and species-specific enzyme activities provides strong correlative evidence linking cross-phase metabolic coordination with furanocoumarin tolerance associated with host-range divergence. Future reciprocal genetic validation, such as allele replacement or transgenic complementation of *H. assulta* with the *CYP6AE19* and key *UGT33* genes from *H. armigera*, would further strengthen this conclusion by directly testing whether restoration of pathway activity increases xanthotoxin metabolism and tolerance.

In summary, our study reveals that the expanded and dynamically diversified UGT33 family constitutes a crucial Phase II detoxification system in *Helicoverpa*, with key roles in the metabolism of specific furanocoumarins in *H. armigera*. In this species, CYP6AE19-mediated O-dealkylation and hydroxylation of xanthotoxin generate glycosylatable intermediates that are subsequently conjugated by UGT33 enzymes, establishing a coordinated cytochrome P450–UGT detoxification cascade. Comparative analysis with the oligophagous *H. assulta* further shows that impairment of this CYP6AE19–UGT33 pathway is associated with reduced xanthotoxin detoxification capacity and high sensitivity to this plant defensive compound. Collectively, these findings provide mechanistic insight into how coordinated Phase I–Phase II metabolism enables insects to detoxify a plant defensive compound and demonstrate that functional divergence of this pathway contributes to interspecific differences in toxin tolerance, which are associated with contrasting host ranges in closely related herbivorous insects.

## Methods

### Insects

The wild-type strain Ha-SCD of *H. armigera* was kindly provided by Prof. Yidong Wu (Nanjing Agricultural University). This strain was originally collected from Côte D’Ivoire, Africa, in the 1970s and has since been maintained in the laboratory without exposure to insecticides. Based on strain Ha-SCD, a series of knockout lines were generated, including SCD-d33c1, SCD-d33c2, SCD-d33c1c2, SCD-d6AE19, SCD-d6AE19-d33c2, and SCD-d6AE-d33c2. The SCD-d33c1 strain carries a deletion encompassing a cluster of eight *UGT33* family genes (*UGT33F2Av2*, *UGT33F2Av1*, *UGT33M1*, *UGT33F3*, *UGT33F1A*, *UGT33F1B*, *UGT33F2B*, and *UGT33B4*), hereafter referred to as cluster 1 (33c1). The SCD-d33c2 strain is deficient in a cluster of 12 *UGT33* genes (*UGT33J1*, *UGT33B1B*, *UGT33B1A*, *UGT33B5*, *UGT33B7A*, *UGT33B7C*, *UGT33B12*, *UGT33B3*, *UGT33B2*, *UGT33B11*, *UGT33B9A*, and *UGT33B8*), referred to as cluster 2 (33c2). The SCD-d33c1c2 strain contains deletions of both 33c1 and 33c2 clusters, comprising a total of 20 *UGT33* genes. The SCD-d6AE strain, derived from our previous work, lacks a cluster of nine *CYP6AE* genes (*CYP6AE14*, *CYP6AE20*, *CYP6AE19*, *CYP6AE18*, *CYP6AE17*, *CYP6AE16*, *CYP6AE11*, *CYP6AE15*, and *CYP6AE12*). The SCD-d6AE19-d33c2 strain harbours deletions of the 12 *UGT33* genes within cluster 2 and a truncated, non-functional *CYP6AE19* protein caused by a 650 bp deletion in exon 1 of the *CYP6AE19* gene. The SCD-d6AE-d33c2 strain carries deletions in both the nine *CYP6AE* genes and the 12 *UGT33* genes of cluster 2.

The Ha-96S strain of *H. armigera* was originally derived from field samples collected in Xinxiang (Henan, China) in 1996 and subsequently inbred for 10 generations by successive single-pair sibling mating. This strain was kindly provided by Prof. Kongming Wu and Gemei Liang (Chinese Academy of Agricultural Sciences).

The Hs-XC strain of *H. assulta* was collected from tobacco fields at the Xuchang campus of Henan Agricultural University and has been reared continuously for more than 10 generations in the laboratory. This strain was kindly provided by Zhongyuan Deng (Zhengzhou University).

Larvae of all strains were reared on an artificial diet based on wheat germ and soybean powder under conditions of 26 ± 1 ^°^C, 60 ± 10% relative humidity (RH) and a photoperiod of 16 h light: 8 h dark. A 10% sugar solution was supplied to adults.

### Chemicals

Xanthotoxin (98%) and flavone (98%) were purchased from Aladdin Biochemical Technology Co., Shanghai, China. Gossypol acetate (98%) was purchased from Shanghai Yuanye Bio-Technology Co., China. DIMBOA (98%), imperatorin (98%) and xanthotoxol glucoside (98%) were purchased from Wuhan Jonk Biological Technology Co., Hubei, China. Bergapten (98%) and xanthotoxol (98%) were purchased from Bide Pharmaceutical Technology Co., Shanghai, China. 5-hydroxyxanthotoxin (98.5%), isoimperatorin (98%) and nicotine (98%) were purchased from PUSH Bio-Technology Co., Chengdu, China. UDP-glucose (98%) was purchased from Sigma-Aldrich. 1-naphthol (1-NA) (99.8%) was purchased from MedChemExpress.

### CRISPR/Cas9 knockout

Previously described methods were used for the design and synthesis of sgRNAs [13]. Briefly, according to the target sequence principle of 5′-N20NGG-3′ (the PAM sequence is underlined), all sgRNAs were designed using CRISPRdirect (http://crispr.dbcls.jp/). Oligonucleotide primers used to synthesise sgRNA templates are shown in S3 Table. sgRNAs were synthesised according to the manufacturer’s instruction (GeneArt Precision gRNA Synthesis Kit, Thermo Fisher Scientific, Pittsburgh, PA).

Before microinjection, fresh eggs laid within 2 hours on gauze were washed with 1% sodium hypochlorite solution and rinsed with distilled water. After being filtered, eggs were placed on a microscope slide (fixed with double-sided tape). About one nanoliter mix of sgRNA and Cas9 protein (GeneArt Platinum Cas9 Nuclease, Thermo Fisher Scientific, Shanghai, China) were injected into individual eggs using a FemtoJet and InjectMan NI 2 microinjection system (Eppendorf, Hamburg, Germany). Injected eggs were placed at 26 ± 1 °C, 60 ± 10% RH for hatching.

According to the positions and orientations of UGT genes on the chromosomes, sgRNA-33J1 and sgRNA-33B8 were designed to target *UGT33J1* and *UGT33B8* at each end of the 33c2 cluster, respectively (Fig 2B). The two sgRNAs, together with Cas9 protein, were co-injected into early embryos of the *H. armigera* Ha-SCD strain. Hatched larvae after injection were reared on an artificial diet to pupation and subsequently to the adult stage (G₀). Thirty-one single pairs of randomly selected G₀ adults were allowed to mate and produce fertile offspring (G_1_). Ten second-instar larvae from each of the G_1_ single-pair families were pooled for genomic DNA extraction. PCR amplification with the specific primer pair J1F/B8R revealed a ~400 bp fragment in the G_1_ progeny from three of the single-pair families. Sequencing of this fragment confirmed the presence of deletion events of 33c2. Two single-pair families carrying distinct deletion types (designated c2T1 and c2T2) were retained, and their larvae were reared to pupa (G_1_). Individual G_1_ were genotyped for the 33c2 deletion using DNA extracted from the last-instar larval exuviae. Among 96 individuals examined from the respective single-pair family, 13 females and 13 males carried the c2T1 deletion, while 16 females and 8 males carrying the c2T2 deletion were identified from 144 individuals of their corresponding single-pair family. Female G_1_ moths carrying the c2T1 deletion were then crossed with male moths carrying the c2T2 deletion (and vice versa) to produce G_2_ offspring (Fig 2B and 2C). When the G_2_ progeny reached the pupal stage, genomic DNA was extracted from the last-instar larval exuviae, and a molecular detection method was developed to distinguish homozygous mutants, heterozygotes, and wild-type individuals (Fig 2B). Homozygous mutants (c2T1/c2T2) displayed characteristic “dual peaks” near the sgRNA target sites in Sanger sequencing chromatograms, corresponding to the superimposed c2T1 and c2T2 patterns. Heterozygotes showed a single c2T1 or c2T2 pattern, whereas wild-type individuals lacked the target amplicon. Homozygous mutant adults were subsequently mass-crossed to generate the G_3_ generation. After oviposition, genomic DNA was extracted from the G_2_ adults, and PCR amplification using the specific primer pair J1F/B8R followed by Sanger sequencing confirmed that all individuals were of the c2T1/c2T2 genotype. To further verify that the G_3_ was composed exclusively of homozygous knockout individuals, a PCR-based genotyping assay was performed. Eight pooled samples (10 early second-instar larvae per pool; 80 larvae in total) were tested using primer pairs J1F/B8R, B1Bf/B1Br, and B9Af/B9Ar. All samples produced the expected amplicon with J1F/B8R, but no product with either B1Bf1/B1Br1 or B9Af/B9Ar, confirming complete homozygosity of the 33c2 knockout (S15B Fig). The resulting stable homozygous deletion strain was designated SCD-d33c2. Following a similar construction procedure, the UGT33 cluster 1 knockout strain was generated and designated SCD-d33c1 (S13 and S15A Figs). Furthermore, based on the SCD-d33c2 strain, the UGT33 cluster 1 was subsequently knocked out to construct a double-cluster knockout strain, which was designated SCD-d33c1c2 (S14 and S15D Fig).

Based on the SCD-d33c2 strain, the *CYP6AE19* gene was further knocked out (S21 Fig). Two sgRNAs targeting the first exon of *CYP6AE19* and Cas9 protein were mixed and injected into freshly laid eggs of the *H. armigera* SCD-d33c2 strain. The hatched larvae were reared to adulthood (G_0_). Genomic DNA was extracted from the forelegs of individual G_0_ adults, and genotyping was conducted using the specific primer pair 19F/R. The PCR product of the wild-type allele was 1,161 bp, whereas the expected amplicon size for deletions between the two sgRNA target sites was approximately 500 bp. Among 24 G_0_ females, 9 individuals showed both 1,161 bp and ~500 bp bands, while 10 such individuals were identified among 32 G_0_ males. Nine single-pair crosses were established from these positive individuals. Progeny (G_1_ larvae) from three of these single-pair families were pooled and reared to adulthood. Genomic DNA was extracted from the forelegs of the G_1_ adults and genotyped using the 19F/R primer pair. Among 32 males and 34 females examined, four males and four females showed only the ~500 bp band. Sanger sequencing of the ~500 bp fragment confirmed that these individuals carried deletions between the two sgRNA target sites. Two distinct edited haplotypes were identified: d6AE19T1, characterised by a 674 bp deletion with a 4 bp insertion (net indel of 670 bp), and d6AE19T2, characterised by a 650 bp deletion (S21B Fig). The G_1_ individuals carrying the d6AE19T1 and d6AE19T2 alleles were intercrossed to produce the G_2_ generation. Genomic DNA was extracted from pooled samples of 10 second-instar G_2_ larvae for PCR analysis. Gel electrophoresis revealed a single ~500 bp band, confirming that all individuals were homozygous for the deletion. The resulting stable homozygous knockout strain was designated SCD-d6AE19-d33c2 (S21C Fig).

To generate a double-knockout strain lacking both the CYP6AE gene cluster and UGT33 cluster 2, female SCD-d6AE moths were mass-crossed with male SCD-d33c2 moths (and vice versa) to produce heterozygous G_1_ individuals, which were subsequently intercrossed to obtain the G_2_ offspring (S20C Fig). For genotyping G_2_ individuals, genomic DNA was extracted from the forelegs of moths prior to mating. Individuals were identified as homozygous knockouts for both the CYP6AE cluster and the UGT33c2 cluster if PCR amplification produced a band with primer pair 14F/12R but no bands with 20f/20r or 15f/15r, and simultaneously produced a band with J1F/B8R but no bands with B1Bf2/B1Br2 or B2f/B2r (S20A and S20B Fig). No female homozygous individuals were obtained in the G_2_ generation. Therefore, male (d6AE/d6AE; d33c2/d33c2) and female (d6AE/d6AE; d33c2/+) individuals were selected and intercrossed to produce G_3_ offspring. Among 109 G_3_ individuals genotyped, 48 were identified as homozygous mutants. These homozygous mutants were then mass-crossed to establish a stable double-knockout strain, which was designated SCD-d6AE-d33c2 (S20 Fig).

### Bioassays of phytochemical toxins

All bioassays with *H. armigera* and *H. assulta* were conducted under controlled environmental conditions of 26 ± 1 °C, 60 ± 10% RH and a 16 h light: 8 h dark photoperiod. The toxicity of phytochemicals was assessed using diet incorporation bioassays based on Wang *and colleagues* [13]. Briefly, unfed neonates (<24 h post-hatching) were individually placed on artificial diet mixed with different concentrations of phytochemicals and untreated artificial diet as a control. A total of 24 larvae were used as a biologically independent sample and 3–4 replicates of each strain were tested for each concentration. Mortality was recorded after 7 days for *H. armigera* and after 8 days for *H. assulta*, and larvae were considered dead if they had died or failed to reach the third instar by the end of the assay. Corrected mortality was calculated using the formula: Corrected mortality = (Mortality in response to treatment − Mortality in response to control)/(1 − Mortality in response to control). Differences in corrected mortality between two strains at each concentration were analysed using two-sided Student’s *t*-*t*ests, whereas comparisons involving three strains were analysed using one-way ANOVA followed by Tukey’s HSD test for multiple pairwise comparisons (*p* < 0.05). To estimate the LC_50_ values for xanthotoxin, seven to 10 concentrations of xanthotoxin were used to establish log-probit lines. Diet incorporation bioassays were executed as described above. The LC_50_ values along with their 95% fiducial limits were calculated via probit analysis using the *PoloPlus* software [46]. Two LC_50_ values were considered significantly different when their 95% fiducial limits did not overlap [47].

### *H. armigera* feeding on artificial diets containing xanthotoxin

To identify the metabolites produced during xanthotoxin metabolism in *H. armigera*, fifth-instar larvae of the Ha-SCD strain were starved for 6 hours and then fed either an artificial diet supplemented with 2 mg/g xanthotoxin or an untreated control diet. Twelve larvae were included per biological replicate, with three independent replicates performed for each treatment. After 24 hours of feeding, frass was collected, pooled, and divided into two 100 mg portions. One portion was subjected to untargeted metabolomic analysis using an Agilent 1290 Infinity LC ultra-high-performance liquid chromatography (UHPLC) system coupled to an AB SCIEX TripleTOF 6600 mass spectrometer. The other portion was analysed by targeted quantification of xanthotoxin, xanthotoxol, 5-hydroxyxanthotoxin, xanthotoxol glucoside, and 5-hydroxyxanthotoxin glucoside using UPLC-MS/MS.

### Heterologous expression of UGT and CYP6AE19 proteins

All recombinant UGTs were expressed using the Bac-to-Bac Baculovirus Expression System (Invitrogen). Briefly, the open reading frames (ORFs) of UGT33 family genes were PCR amplified from the Ha-SCD strain of *H. armigera* and Hs-XC strains of *H. assulta* and cloned into the pFastBacHTC vector (Invitrogen) with In Fusion Snap Assembly cloning kits (Takara Bio, China). Primers for full-length cloning are shown in S3 Table. The ORFs of *HaUGT33B7A*, *HaUGT33B7C* and *HaUGT33B8* were synthesised commercially. Plasmids of recombinants were transformed into *E. coli* DH10Bac competent cells, and the recombinant bacmids were then transfected to Sf9 cells using the FuGENE HD Transfection Reagent (Promega, Madison, Wisconsin). High-titer recombinant baculovirus stocks were infected to Sf9 cells for producing recombinant UGT proteins. Microsomal fractions were prepared using a microsome isolation kit (Abcam) and quantified with a BCA protein assay kit (Thermo Fisher Scientific). The expression of UGTs in the microsomal fractions was subsequently determined by Western blotting using a 6× His-tag monoclonal antibody (1:6,000 dilution; Thermo Fisher Scientific), and an anti-mouse module were used for chemiluminescent detection.

Expression and characterisation of HaCYP6AE19 were performed as described by Wang and colleagues (2018) [18]. For HsCYP6AE19, the ORF was cloned from the Hs-XC strain and expressed in Sf9 cells using the Bac-to-Bac system (Invitrogen), with co-expression of HsCPR at MOIs of 2 and 0.2, respectively. P450 content in microsomal protein was determined by reduced CO-difference spectral assay.

### Activity assays for model substrates

UGT activity was determined against the model substrate 1-naphthol (1-NA). The reactions were performed in a total volume of 100 μl containing 5 μg of crude microsomal UGT protein, 500 μM UDP-glucose, 1 mM MgCl_2_, and 20 μM 1-NA in 0.1 M potassium phosphate buffer (pH 7.4). Following a 5 min pre-incubation at 30 °C, reactions were initiated by the addition of 1-NA and incubated at 30 °C with shaking at 1,200 rpm for 2 h. Reactions were terminated by addition of UDP Detection Reagent from the UDP-Glo Glycosyltransferase Assay (Promega). Negative controls consisted of reaction mixtures lacking 1-NA. The formation of free UDP was quantified using the UDP-Glo Glycosyltransferase Assay, which converts free UDP to ATP, generating light in a luciferase reaction. According to the manufacturer’s protocol, each glycosyltransferase reaction was mixed 1:1 with UDP-Glo Detection Reagent (three technical replicates) and incubated at room temperature for 1 h. Luminescence was then measured in relative luminescence units (RLU). For absolute quantification, a UDP standard curve (0–25 μM UDP) was generated and plotted using Excel software. The range of measurements was confirmed to be within the linear detection range.

### In vitro metabolism of chemicals

In vitro metabolism assays using recombinant UGT enzymes were performed in a total volume of 100 μl containing 5 μg of crude microsomal UGT protein, 500 μM UDP-glucose, 1 mM MgCl_2_, and 20 μM substrate (xanthotoxol or 5-hydroxyxanthotoxin) in 0.1 M potassium phosphate buffer (pH 7.4). Reactions were initiated by the addition of substrate following a 5-min pre-incubation at 30 °C. The mixtures were incubated for 2 hours at 30 °C with shaking at 1,200 rpm. Reactions were stopped by adding 100 μl methanol, followed by dilution with 800 μl of 50% methanol (v/v) and incubation for an additional 20 min. The samples were then centrifuged at 18,000*g* for 10 min, and 200 μl of the supernatant was transferred to HPLC vials for analysis. Reaction mixtures containing microsomal fractions from cells transfected with the empty baculovirus vector served as controls. The formation of xanthotoxol glucoside and the depletion of 5-hydroxyxanthotoxin were used as indicators of enzymatic activity towards xanthotoxol and 5-hydroxyxanthotoxin, respectively.

The in vitro metabolism of xanthotoxin by CYP6AE19 was performed as described by Wang *and colleagues* (2018) [18] with minor modifications. Briefly, reactions were carried out in 400 µL of 0.1 M potassium phosphate buffer (pH 7.4) containing 10.5 pmol recombinant P450, NADPH regeneration system (Promega, Madison, WI), and 40 µM xanthotoxin. After pre-warming at 30 °C for 5 min, the reaction was initiated by xanthotoxin addition and incubated at 30 °C with shaking at 1,200 rpm for 4 h. The reaction was stopped with 400 µL acetonitrile, followed by 1,200 µL dilution buffer (50% potassium phosphate buffer and 50% acetonitrile). After 20 min, the mixture was centrifuged at 18,000*g* for 10 min, and 500 µL of the supernatant was analysed by HPLC. Negative controls (non-insertion microsomes) and samples without NADPH were tested in parallel. The reaction products were analysed using UPLC–MS/MS with targeted detection of xanthotoxin, xanthotoxol and 5-hydroxyxanthotoxin.

### UPLC–MS/MS

Samples were separated by HSS T3 column (2.1 × 100 mm, 1.8 μm particle size, Waters, MA) on UPLC system (Waters ACQUITY UPLC I-Class) with different gradient elution programs, the flow speed was 0.3 ml/min. 4 μl samples were separated with A (water/0.05% (v/v) formic acid) and B (Acetonitrile/0.05% (v/v) formic acid). A gradient elution program was established as follows: 0–1 min, A:B = 90:10; 4 min, A:B = 60:40; 6 min, A:B = 40:60; 7–8 min, A:B = 10:90; and 9–10 min, A:B = 90:10. Samples were detected by tandem quadrupole mass spectrometer (QTRAP 5,500, SCIEX) run in positive ESI mode. The multiple reaction monitoring (MRM) transitions 217.1 > 202.1, 203.1 > 147.1, 233.2 > 218.0, 365.2 > 203.1 and 395.0 > 233.0 were used for the detection of xanthotoxin, xanthotoxol, 5-hydroxyxanthotoxin, xanthotoxol glucoside and 5-hydroxyxanthotoxin glucoside, respectively (S22–S26 Fig). Authentic standards were used to confirm xanthotoxin, xanthotoxol, 5-hydroxyxanthotoxin and xanthotoxol glucoside by comparison of their retention times and MS/MS fragmentation patterns with those of authentic standards. Because no authentic standard was available, 5-hydroxyxanthotoxin glucoside was putatively identified based on the MRM transition *m*/*z* 395.0 > 233.0, corresponding to a neutral loss of 162 Da consistent with the loss of a hexose moiety. UGT33 activity towards 5-hydroxyxanthotoxin was therefore quantified by substrate depletion using the authentic substrate standard.

### Bioinformatic and phylogenetic analysis

UGT genes were identified from chromosome-level reference genomes of *H. armigera* (Ha-SCD, GCA_023701775.1; ASM3070526v1, Ha-96S, GCA_030705265.1; ilHelArmi9.1, Ha-UK, GCA_963930815.1; ASM4095451v1, Ha-GR, GCA_040954515.1), *H. assulta* (GCA_963856015.1), *H. peltigera* (GCA_958496145.1), and *H. viriplaca* (GCA_976234845.1) using the BLAST algorithm within the Geneious software suite (Biomatters, New Zealand), with UGT sequences from *H. armigera* and *S. frugiperda* as query sequences. Genomic regions containing UGT33 genes located were carefully examined to avoid overestimating gene losses. The Multiple alignment, conserved motif [48] and exon/intron organisation were used to verify UGT gene completeness. For phylogenetic analysis, UGT protein sequences were aligned using MUSCLE algorithm [49] within Geneious software suite, and a phylogenetic tree of *H. armigera* UGT proteins was constructed using the Maximum Likelihood method in MEGA 12 [50] with 1,000 bootstraps. The UGT genes in *S. frugiperda* and *S. litura* were obtained from Wang *and colleagues* (2024) [13].

For calculation of sequencing depth, adapters and low-quality bases were removed using fastp v0.23.4 [51], then the clean reads were mapped to the Ha-SCD reference genome [52] using bwa v0.7.19 [53]. The resulting BAM files were sorted and duplicated reads were marked using SAMtools v1.19.2 [54] and Sambamba v 1.0.1 [55], respectively. Finally, the sequencing depth across the entire chromosome and the UGT gene clusters was determined using the inbuilt depth function of SAMtools v1.19.2 [54].

To infer the phylogenetic relationships among species, single-copy orthologous genes were identified using BUSCO v5.4.3 with the lepidoptera_odb10 reference dataset. The script busco2fasta.py (available at https://github.com/lstevens17/busco2fasta) was applied to extract a total of 1,764 shared BUSCO genes shared across all species [56]. For each single-copy orthologue, protein sequences were aligned with MAFFT v7.475 [57], and the corresponding aligned CDS sequences were generated via pal2nal v14. Aligned CDS sequences were concatenated into a supergene using custom Python scripts. The concatenated gene was subsequently used to infer a rooted phylogram in IQ-TREE v2.0.3 [58]. To construct an ultrametric time tree, substitution rates were first estimated with baseml, and divergence times were then calculated using mcmctree; both programs are modules integrated within the PAML v4.9 software package [59]. The divergence time between *S. litura* and *S. frugiperda* was calibrated to 12.45 million years ago (Mya) based on Kergoat and colleagues’s study [60].

To analyse nonsynonymous/synonymous substitution rate ratios (dN/dS), UGT33 protein and corresponding CDS sequences for each species were used. Protein sequences were aligned using MAFFT, and a phylogenetic tree constructed with IQ-TREE. The OrthoSNAP v1.6.0 pipeline was then applied to delineate subfamilies of single-copy orthologous UGT genes [61]. We retained only subfamilies harbouring orthologues from a minimum of four species, yielding a final set of 18 subfamilies for downstream analyses. CDS alignments were generated from the corresponding protein sequence alignments using pal2nal. To estimate non-synonymous (dN) and synonymous (dS) substitutions for each UGT subfamily, we implemented the FitMG94 model in HyPhy v2.5.100 with the parameters --type local --lrt Yes [62]. Pairwise interspecific comparisons of dN/dS ratios were conducted via Mann–Whitney *U* tests, and multiple testing bias was corrected by adjusting raw P-values according to the Benjamini–Hochberg (BH) procedure.

### Determination of relative copy number of UGT33 genes by quantitative PCR

Four candidate UGT genes (*UGT33J1*, *UGT33B5*, *UGT33B3*, and *UGT33M1*) were selected for quantitative PCR (qPCR) analysis to determine their relative copy numbers in the Ha-SCD strain compared to Ha-96S strain. Gene-specific primers were designed in regions identical between the two reference genomes using the Geneious software suite (Biomatters, New Zealand). The *H. armigera* sodium channel gene *para* (XM_049846476.2) served as the internal reference. Primer sequences are listed in S3 Table. Genomic DNA was extracted from 12 third-instar larvae of both Ha-SCD and Ha-96S strains and normalised to equal concentrations for qPCR analysis. Reactions were performed on an ABI 7500 Real-Time PCR System (Applied Biosystems, Foster City, CA, USA) in 20 μl volumes containing 0.4 μM of each primer, 2 μl of DNA template and 10 μl of SYBR Green Master Mix, with DNase-free water added to the final volume. Thermal cycling conditions were: 95 °C for 30 s; followed by 40 cycles of 95 °C for 5 s and 60 °C for 30 s; with a final dissociation step from 60 °C to 95 °C, holding at 95 °C for 15 s. Relative gene copy numbers were calculated using the 2^−ΔΔCT^ method, with the Ha-96S strain used as the calibrator.

### Induction treatment and gene expression analysis

To characterise the expression patterns of UGT genes across developmental stages and host plant diets, RNA-seq analyses were conducted using the H. armigera SCD strain. For developmental-stage profiling, whole-body samples were collected from eggs, second instar larvae, fifth instar larvae, female pupae, male pupae, female adults, and male adults. Three biological replicates were prepared for each developmental stage, with 10 individuals per replicate except for eggs with ~100 individuals per replicate.

To assess host plant-associated expression, newly moulted fifth-instar larvae were allowed to feed for 48 h on leaves and flower buds of large bullwort (*Ammi majus* L.), fruits of kumquat (*Citrus japonica* Thunb. cv. Jingen), leaves of cotton (*Gossypium hirsutum* cv. Jimian 11), seedlings of maize (*Zea mays* cv. B73), or leaves of tobacco (*Nicotiana benthamiana*). Midgut tissues were subsequently dissected and pooled from 10 larvae per biological replicate. Three biological replicates were prepared for each host plant treatment.

To evaluate xanthotoxin-induced transcriptional responses in *H. armigera* and *H. assulta*, newly moulted fifth-instar larvae of each species were fed an artificial diet supplemented with 1 mg/g xanthotoxin for 48 h. Larvae fed an unsupplemented artificial diet served as controls. After treatment, midguts were dissected for RNA extraction. Midguts from 10 larvae were pooled as a biological replicate, and three biological replicates were prepared.

For all RNA-seq samples, total RNA extraction, quality assessment, library preparation and Illumina sequencing were performed by Novogene Technology Co. (Tianjin, China). Raw reads were first filtered using the Quick Run Trimmomatic module [63] in TBtools-II v2.485 [64]. The clean reads were then analysed using the One Step RNAseq 2 Expression module in TBtools-II, which integrates HISAT2 [65], SAMtools [54] and StringTie [66] for read alignment, alignment file processing and transcript/gene expression quantification, respectively. Differentially expressed genes were subsequently identified using the Differential Gene Expression Analysis (DESeq2) module [67] in TBtools-II [64]. For *H. armigera* samples, the Ha-96S genome assembly (GCA_030705265.1) was used as the reference genome, whereas for *H. assulta* samples, the corresponding *H. assulta* genome assembly (GCA_963856015.1) was used. Gene expression levels were visualised as heatmaps using TBtools-II [64].

## Supporting information

S1 Fig Genomic position of UGT genes *in H. armigera.* The genomic distribution of UGT genes is shown based on the *H. armigera* SCD (Ha-SCD) genome assembly. The Ha-SCD strain, originally collected from Côte d’Ivoire (Ivory Coast, Africa), served as the reference background for this study. Genes belonging to the three largest UGT families (UGT33, UGT40 and UGT41) are highlighted in red, green, and blue, respectively. The UGT33 family is organised into two major gene clusters on chromosome 28, comprising eight and 12 genes, respectively, with the exception of *UGT33T1*, which is located on chromosome 9. Misannotated copies of UGT33 family genes are shown in grey. Yellow highlighting indicates partial or pseudogene UGT.
(PDF)

S2 Fig Genomic position of UGT genes *in H. armigera.* The genomic distribution of UGT genes is shown based on the *H. armigera* 96S (Ha-96S) genome assembly. The Ha-96S strain was originally collected from Henan Province, China, Asia. Genes belonging to the three largest UGT families (UGT33, UGT40 and UGT41) are highlighted in red, green, and blue, respectively. The UGT33 family is organised into two major gene clusters on chromosome 24, comprising seven and 12 genes, respectively, with the exception of *UGT33T1*, which is located on chromosome 12.
(PDF)

S3 Fig Genomic position of UGT genes *in H. armigera.* The genomic distribution of UGT genes is shown based on the *H. armigera* GR (Ha-GR) genome assembly. The Ha-GR strain was originally collected from Canberra, Australia, Oceania. Genes belonging to the three largest UGT families (UGT33, UGT40 and UGT41) are highlighted in red, green, and blue, respectively. The UGT33 family is organised into two major gene clusters on chromosome 28, comprising eight and 14 genes, respectively, with the exception of *UGT33T1*, which is located on chromosome 9.
(PDF)

S4 Fig Genomic position of UGT genes *in H. armigera.* The genomic distribution of UGT genes is shown based on the *H. armigera* UK (Ha-UK) genome assembly. The Ha-UK strain was originally collected from United Kingdom, Europe. Genes belonging to the three largest UGT families (UGT33, UGT40 and UGT41) are highlighted in red, green, and blue, respectively. The UGT33 family is organised into two major gene clusters on chromosome 24, comprising nine and 12 genes, respectively, with the exception of *UGT33T1*, which is located on chromosome 7.
(PDF)

S5 Fig Genomic position of UGT genes *in H. assulta.* Genes belonging to the three largest UGT families (UGT33, UGT40 and UGT41) are highlighted in red, green, and blue, respectively. Yellow highlighting indicates partial or pseudogene UGT.
(PDF)

S6 Fig Genomic position of UGT genes in *Heliothis viriplaca.* Genes belonging to the three largest UGT families (UGT33, UGT40 and UGT41) are highlighted in red, green, and blue, respectively. Yellow highlighting indicates partial or pseudogene UGT.
(PDF)

S7 Fig Genomic position of UGT genes in *Heliothis peltigera.* Genes belonging to the three largest UGT families (UGT33, UGT40 and UGT41) are highlighted in red, green, and blue, respectively.
(PDF)

S8 Fig Relative genomic copy numbers of four representative UGT33 family genes in *H. armigera* SCD compared with the Ha-96S strain, determined by quantitative PCR. The sodium channel gene (*para*, XM_049846476.2) was used as a single-copy internal reference. Relative gene copy numbers were calculated using the 2^–ΔΔCT^ method, with the Ha-96S strain serving as the calibrator. The data underlying this figure can be found at S1 Data.
(TIF)

S9 Fig Exonic structures of the gene loci corresponding to *UGT33F2A* and *UGT46A3.* Both *UGT33F2A* (v1 and v2) and *UGT46A3* (v1 and v2) possess distinct exon 1 but share identical exons 2–4. The N-terminal half of the proteins (∼280aa), which is thought to mediate substrate recognition, is encoded by exon 1 in both *UGT33F2A* and *UGT46A3*. **(A, C)** Exonic structures and lengths are drawn to scale according to their actual genomic positions on the chromosome. **(B, D)** Exon lengths are drawn to scale, with introns omitted for clarity.
(TIF)

S10 Fig Distribution of dN (nonsynonymous substitution rate)/dS (synonymous substitution rate) ratios for UGT genes. Pairwise comparisons were conducted via Mann–Whitney *U* tests, with *P*-values corrected by the BH method. ns, not significant; * *P* < 0.05. The data underlying this figure can be found at S1 Data.
(PDF)

S11 Fig Phylogenetic tree with 1,764 shared BUSCO genes from 9 species/strains to show divergence times. The data underlying this figure can be found at S1 Data.
(TIF)

S12 Fig Transcriptomic analysis of *H. armigera* UGT genes. **(A)** Phylogeny of *H. armigera* UGT genes. UGTs were aligned with the outgroup Fringe in MEGA 12 using MUSCLE. Phylogeny was estimated using the Maximum Likelihood method in MEGA 12 with 1,000 bootstraps (substitution model LG+G+I). **(B)** Tissue-specific transcriptomic profiling of *H. armigera* UGT genes across different developmental stages and under different dietary conditions. The heat map was generated using log_2_ [FPKM (Fragments Per Kilo‑base of gene per Million mapped fragments) + 1] values. The colour and size of circles indicate the expression level of each UGT gene in each sample. The data underlying this figure can be found at S1 Data.
(TIF)

S13 Fig CRISPR-Cas9-mediated knockout of UGT33 cluster 1 in the *H. armigera* SCD strain, resulting in the SCD-d33c1 strain. **(A)** The UGT genes are shown in their correct orientation and order, although gene lengths and intergenic distances are not to scale. **(B)** Representative sequencing chromatograms illustrating the construction of SCD-d33c1 strain. The target sequences of the sgRNAs are shown in blue, and the PAM sequences in red. c1T1 and c1T2 represent two distinct genotypes generated by the deletion of an approximately 44-kb genomic fragment between two sgRNAs targeting *UGT33F2Av2* and *UGT33B4*, respectively. These genotypes resulted from imprecise DNA repair through the non-homologous end joining (NHEJ) pathway following CRISPR-Cas9–induced double-strand breaks. c1T1/c1T2 represents overlapping chromatogram peaks of c1T1 and c1T2 within a single individual, indicating the coexistence of both genotypes and confirming a homozygous knockout of the UGT33 cluster 1.
(TIF)

S14 Fig CRISPR-Cas9-mediated knockout of UGT33 cluster 1 in the *H. armigera* SCD-d33c2 strain, resulting in the SCD-d33c1c2 strain. **(A)** The UGT genes are shown in their correct orientation and order, although gene lengths and intergenic distances are not to scale. **(B)** Representative sequencing chromatograms illustrating the construction of SCD-d33c1c2. The target sequences of the sgRNAs are shown in blue, and the PAM sequences in red. c1T3 represent the genotypes generated by the deletion of an approximately 44-kb genomic fragment between two sgRNAs targeting *UGT33F2Av2* and *UGT33B4*, respectively. The genotypes resulted from imprecise DNA repair through the non-homologous end joining (NHEJ) pathway following CRISPR-Cas9–induced double-strand breaks.
(TIF)

S15 Fig CRISPR-Cas9-mediated individual and combined knockout of two UGT33 gene clusters in *H. armigera.* **(C)** Positions of sgRNAs and primer pairs used for allele-specific PCR detection. **(A, B, D)** Genotyping of individual *H. armigera* for deletion of the UGT cluster according to banding patterns of the PCR products amplified with a set of primer pairs. The PCR bands amplified by primer pairs shown in blue spanning two sgRNAs represent deletion events of UGT clusters. The PCR bands amplified by primer pairs shown in red within the UGT cluster indicate the presence of UGT genes in the cluster. Individuals with PCR bands amplified by the primer pairs shown in blue and without a band amplified by the primer pairs shown in red were considered homozygous mutant for the UGT gene cluster knockout.
(TIF)

S16 Fig Metabolite profiling of frass from *H. armigera* larvae fed on xanthotoxin-supplemented or control artificial diets. SCD_XAT, frass from larvae fed on xanthotoxin-supplemented artificial diet; SCD_AD, frass from larvae fed on the control artificial diet without xanthotoxin. **(A)** Fold-change analysis of differential metabolites in positive ion mode by high-resolution untargeted metabolomics. **(B)** Targeted UPLC–MS/MS detection of xanthotoxin and its potential metabolites, including xanthotoxol, 5-hydroxyxanthotoxin, xanthotoxol glucoside, and 5-hydroxyxanthotoxin glucoside. The data underlying this figure can be found at S1 Data.
(TIF)

S17 Fig Representative western blots showing immunoreactive UGT33 family proteins. Twenty-three UGT33 proteins were heterologously expressed in insect cells using the baculovirus expression system.
(TIF)

S18 Fig Activity of recombinant UGT33 proteins from *H. armigera* and *H. assulta* towards the model substrate 1-naphthol (1-NA). UGT activity was measured by quantifying the release of free UDP after incubation of recombinant UGT enzymes with 1-NA. Data are means ± SEM (*n* = 3). Statistical significance was determined by unpaired *t*-tests comparing each recombinant UGT protein with the empty-vector control, *P < 0.05, **P < 0.01, ***P < 0.001. The data underlying this figure can be found at S1 Data.
(TIF)

S19 Fig Reduced CO-difference spectrum of the recombinant HsCYP6AE19. The data underlying this figure can be found at S1 Data.
(TIF)

S20 Fig Generation of the *H. armigera* SCD-d6AE-d33c2 strain. **(A)** Positions of primer pairs used for allele-specific PCR detection. **(B)** Genotyping of individual *H. armigera* for simultaneous deletions in both the CYP6AE and UGT33 cluster 2. Homozygous mutants were identified by the presence of bands generated with all blue-labelled primers and the absence of bands generated with red-labelled primers. **(C)** Construction scheme of the SCD-d6AE-d33c2 strain.
(TIF)

S21 Fig Generation of the *H. armigera* SCD-d6AE19-d33c2 strain. This strain was generated by knocking out the *CYP6AE19* gene based on the SCD-d33c2 background. **(A)** Schematic representation of the *CYP6AE19* gene structure and sgRNA design. sgRNA target sequences and PAM sites are highlighted in blue and red, respectively. **(B)** Representative Sanger sequencing chromatograms show two distinct PCR amplicons flanking the sgRNA target region in the *CYP6AE19* locus, both predicted to produce truncated, non-functional proteins. **(C)** Genotyping of individual *H. armigera* for targeted deletions in the *CYP6AE19* gene and the UGT33 cluster 2. For *CYP6AE19*, PCR products of wild-type and knockout individuals were 1,161 bp and approximately 500 bp, respectively, amplified using the 19F/19R primer pair. For *UGT33* cluster 2, knockout individuals showed a band with primers J1F/B8R but no bands with primer pairs B1Bf2/B1Br2 and B2f/B2r.
(TIF)

S22 Fig Representative MS/MS product ion spectrum (upper) and MRM transition (217.1 → 202.1) (lower) of xanthotoxin detected by UPLC–MS/MS. The data underlying this figure can be found at S1 Data.
(TIF)

S23 Fig Representative MS/MS product ion spectrum (upper) and MRM transition (203.1 → 147.1) (lower) of xanthotoxol detected by UPLC–MS/MS. The data underlying this figure can be found at S1 Data.
(TIF)

S24 Fig Representative MS/MS product ion spectrum (upper) and MRM transition (233.2 → 218.0) (lower) of 5-hydroxyxanthotoxin detected by UPLC–MS/MS. The data underlying this figure can be found at S1 Data.
(TIF)

S25 Fig Representative MS/MS product ion spectrum (upper) and MRM transition (365.2 → 203.1) (lower) of xanthotoxol glucoside detected by UPLC–MS/MS. The data underlying this figure can be found at S1 Data.
(TIF)

S26 Fig Representative MRM transition (395.0 → 233.0) of 5-hydroxyxanthotoxin glucoside detected by UPLC–MS/MS. The data underlying this figure can be found at S1 Data.
(TIF)

S27 Fig Expression profiles of UGT and *CYP6AE19* genes in *H. armigera* and *H. assulta* after xanthotoxin exposure. Expression levels are shown as log_2_ (FPKM + 1), where FPKM represents fragments per kilobase of transcript per million mapped reads. The colour and size of circles indicate the corresponding gene. Asterisks indicate genes that are absent from *H. assulta*. The data underlying this figure can be found at S1 Data.
(TIF)

S28 Fig Differentially expressed UGT and CYP6AE19 genes in *H. armigera* Ha-SCD and *H. assulta* Hs-XC after xanthotoxin exposure. Differentially expressed genes were identified using an adjusted *P* value (padj) < 0.05 and an absolute log_2_Fold Change > 1 as thresholds. The Venn diagram shows genes differentially expressed specifically in Ha-SCD, specifically in Hs-XC, and shared between the two species. Genes highlighted in pink were upregulated after xanthotoxin exposure, genes highlighted in blue were downregulated, and genes highlighted in green were upregulated in Ha-SCD but downregulated in Hs-XC. *CYP6AE19* and UGT33 genes are highlighted in bold and enlarged font. The data underlying this figure can be found at S1 Data.
(TIF)

S1 Table Resequencing of the *H. armigera* SCD strain genome. The sequencing depth across the entire chromosome 28 and the UGT gene clusters was determined using the inbuilt depth function of Samtools.
(DOCX)

S2 Table Sensitivity of newly hatched larvae of different strains to xanthotoxin. (DOCX)

S3 Table PCR primers used in this study. (DOCX)

S4 Table Biological characteristics of the SCD-d33c2 strain and its parental Ha-SCD strain of *H. armigera.* (DOCX)

S1 Data Raw numerical data for each figure. (XLSX)

S2 Data UGT sequences in this study. (XLSX)

S1 Raw images Original western blot images. (PDF)

S2 Raw images Original agarose gel images. (PDF)

## Abbreviations

BH: Benjamini–Hochberg
MRM: multiple reaction monitoring
Mya: million years ago
NHEJ: non-homologous end joining
ORFs: open reading frames
PSMs: plant secondary metabolites
RH: relative humidity
RLU: relative luminescence units
UGT: UDP-glycosyltransferase
UHPLC: ultra-high-performance liquid chromatography
