# Supplementary material for: Coordinated P450–UGT detoxification contributes to furanocoumarin tolerance associated with host plant range divergence in Helicoverpa
Source: PLoS Biol. 2026 Sep 22;24(9):e3004013. doi: 10.1371/journal.pbio.3004013 (PMC13623160; doi:10.1371/journal.pbio.3004013)
Supplement: Supplementary file 2 [file pbio.3004013.s002.pdf]

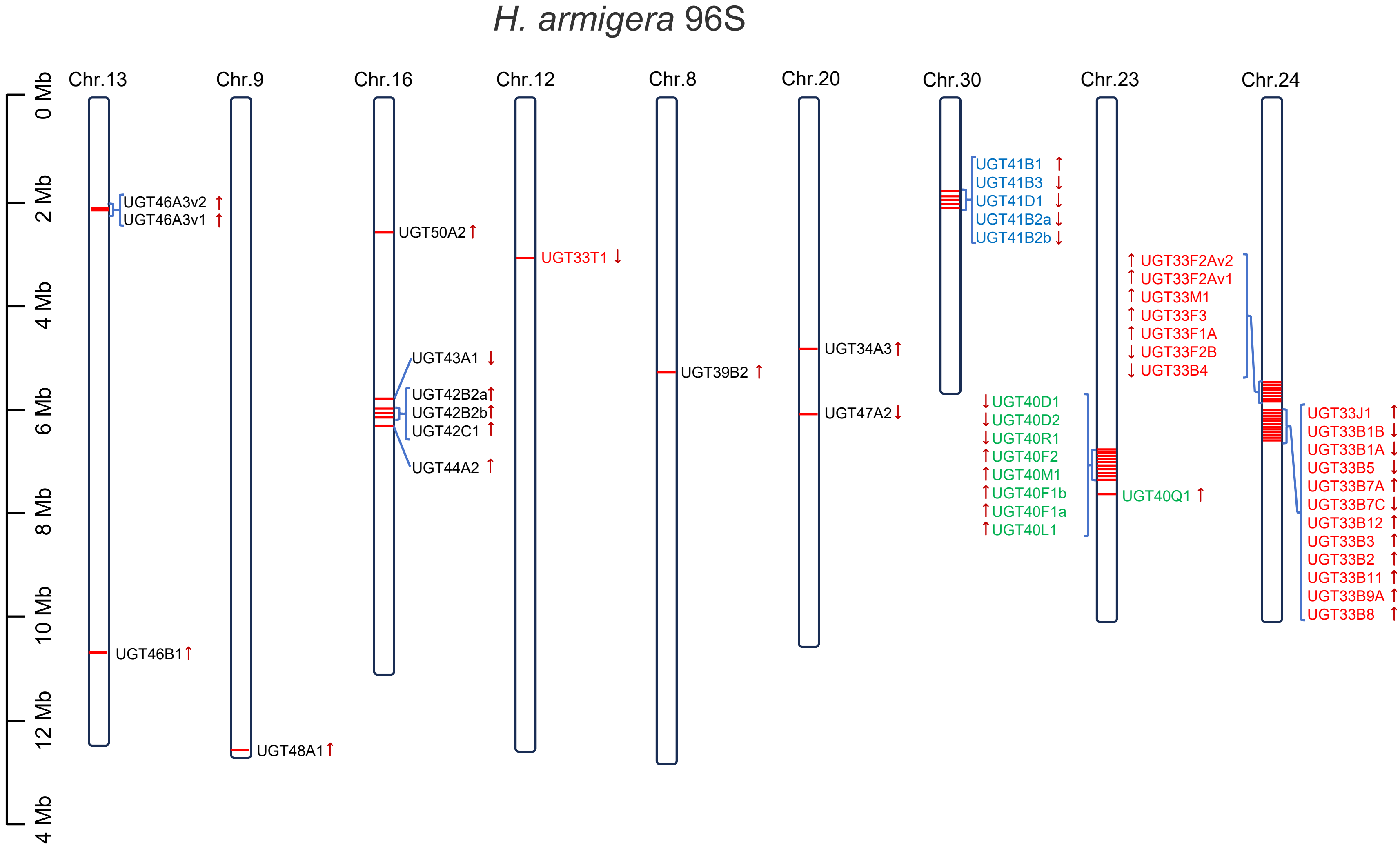

H. armigera 96S
Chr.13
Chr.9
Chr.16
Chr.12
Chr.8
Chr.20
Chr.30
Chr.23
Chr.24
0 Mb
2 Mb
4 Mb
6 Mb
8 Mb
10 Mb
12 Mb
14 Mb
UGT46A3v2 ↑
UGT46A3v1 ↑
UGT46B1↑
UGT48A1↑
UGT50A2↑
UGT43A1 ↓
UGT42B2a↑
UGT42B2b↑
UGT42C1 ↑
UGT44A2 ↑
UGT33T1 ↓
UGT39B2 ↑
UGT34A3↑
UGT47A2↓
UGT41B1 ↑
UGT41B3 ↓
UGT41D1 ↓
UGT41B2a↓
UGT41B2b↓
↓UGT40D1
↓UGT40D2
↓UGT40R1
↑UGT40F2
↑UGT40M1
↑UGT40F1b
↑UGT40F1a
↑UGT40L1
UGT40Q1 ↑
↑ UGT33F2Av2
↑ UGT33F2Av1
↑ UGT33M1
↑ UGT33F3
↑ UGT33F1A
↓ UGT33F2B
↓ UGT33B4
UGT33J1 ↑
UGT33B1B ↓
UGT33B1A ↓
UGT33B5 ↓
UGT33B7A ↑
UGT33B7C ↓
UGT33B12 ↑
UGT33B3 ↑
UGT33B2 ↑
UGT33B11 ↑
UGT33B9A ↑
UGT33B8 ↑
