# Supplementary material for: Coordinated P450–UGT detoxification contributes to furanocoumarin tolerance associated with host plant range divergence in Helicoverpa
Source: PLoS Biol. 2026 Sep 22;24(9):e3004013. doi: 10.1371/journal.pbio.3004013 (PMC13623160; doi:10.1371/journal.pbio.3004013)
Supplement: Supplementary file 3 [file pbio.3004013.s003.pdf]

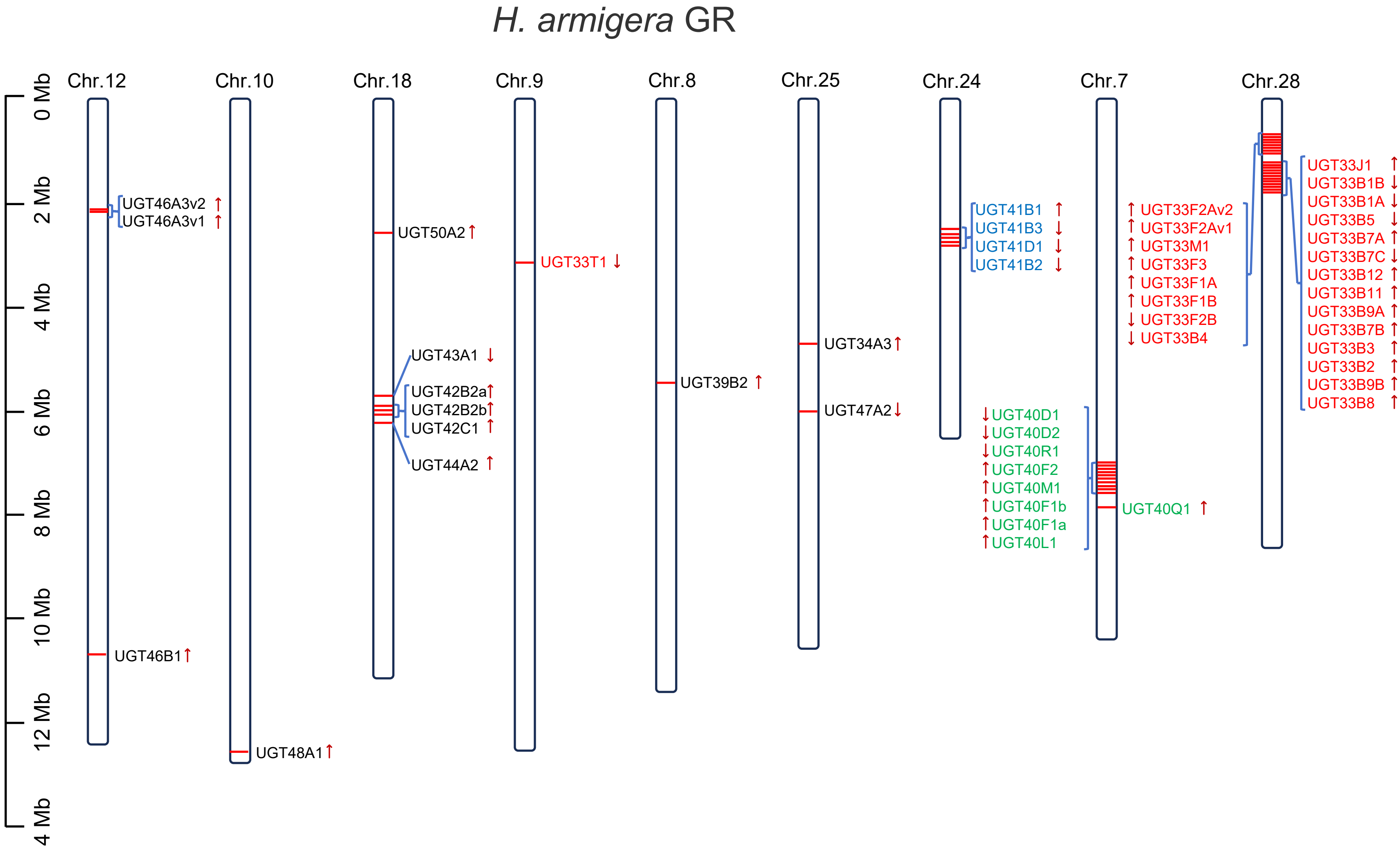

H. armigera GR
0 Mb
2 Mb
4 Mb
6 Mb
8 Mb
10 Mb
12 Mb
14 Mb
Chr.12
Chr.10
Chr.18
Chr.9
Chr.8
Chr.25
Chr.24
Chr.7
Chr.28
UGT46A3v2
UGT46A3v1
UGT46B1
UGT48A1
UGT50A2
UGT43A1
UGT42B2a
UGT42B2b
UGT42C1
UGT44A2
UGT33T1
UGT39B2
UGT34A3
UGT47A2
UGT41B1
UGT41B3
UGT41D1
UGT41B2
UGT40D1
UGT40D2
UGT40R1
UGT40F2
UGT40M1
UGT40F1b
UGT40F1a
UGT40L1
UGT40Q1
UGT33F2Av2
UGT33F2Av1
UGT33M1
UGT33F3
UGT33F1A
UGT33F1B
UGT33F2B
UGT33B4
UGT33J1
UGT33B1B
UGT33B1A
UGT33B5
UGT33B7A
UGT33B7C
UGT33B12
UGT33B11
UGT33B9A
UGT33B7B
UGT33B3
UGT33B2
UGT33B9B
UGT33B8
