# Supplementary material for: Coordinated P450–UGT detoxification contributes to furanocoumarin tolerance associated with host plant range divergence in Helicoverpa
Source: PLoS Biol. 2026 Sep 22;24(9):e3004013. doi: 10.1371/journal.pbio.3004013 (PMC13623160; doi:10.1371/journal.pbio.3004013)
Supplement: Supplementary file 6 [file pbio.3004013.s006.pdf]

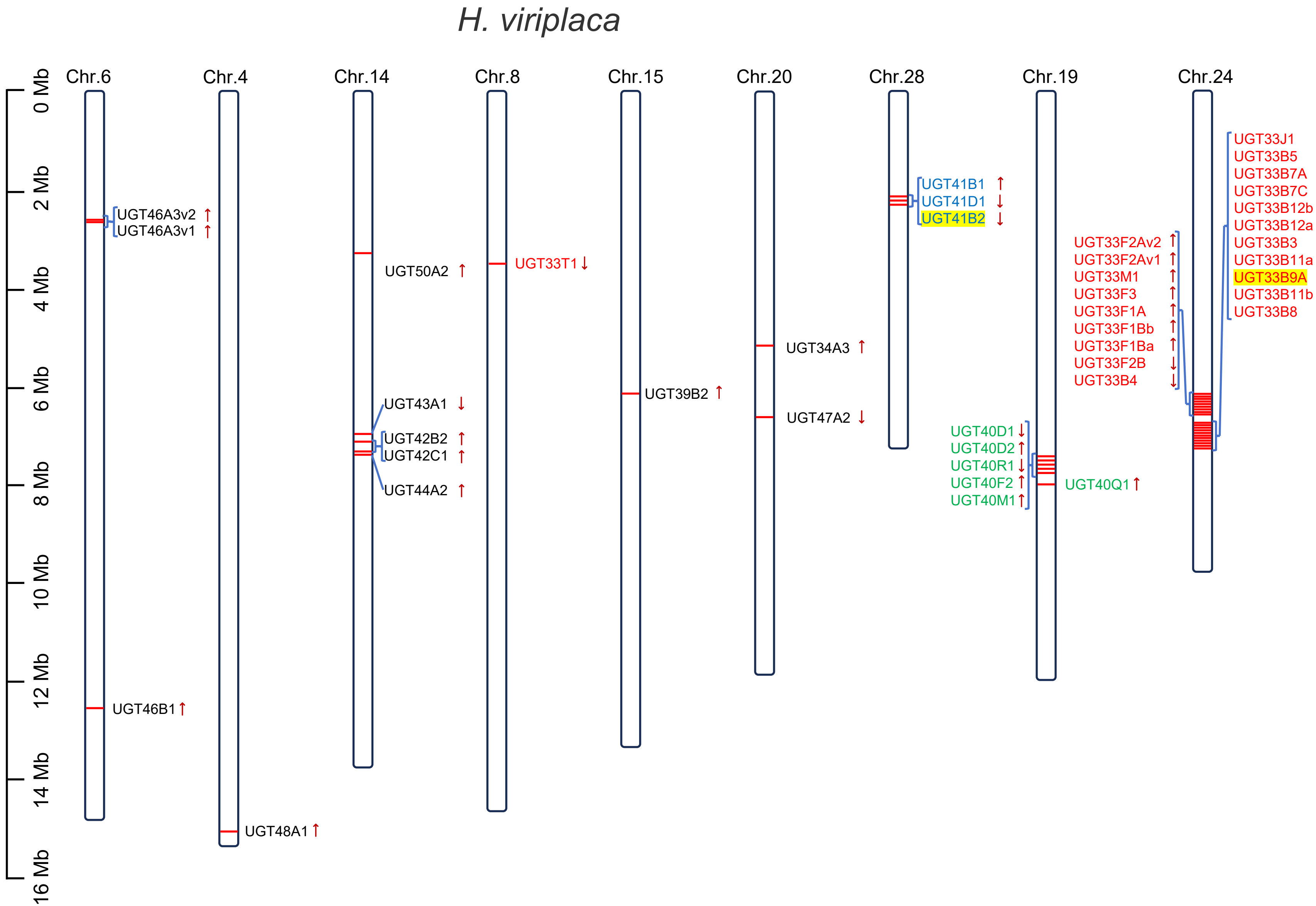
H. viriplaca
0 Mb
2 Mb
4 Mb
6 Mb
8 Mb
10 Mb
12 Mb
14 Mb
16 Mb
Chr.6
UGT46A3v2 ↑
UGT46A3v1 ↑
UGT46B1 ↑
Chr.4
UGT48A1 ↑
Chr.14
UGT50A2 ↑
UGT43A1 ↓
UGT42B2 ↑
UGT42C1 ↑
UGT44A2 ↑
Chr.8
UGT33T1 ↓
Chr.15
UGT39B2 ↑
Chr.20
UGT34A3 ↑
UGT47A2 ↓
Chr.28
UGT41B1 ↑
UGT41D1 ↓
UGT41B2 ↓
Chr.19
UGT40D1 ↓
UGT40D2 ↑
UGT40R1 ↓
UGT40F2 ↑
UGT40M1 ↑
UGT40Q1 ↑
Chr.24
UGT33F2Av2 ↑
UGT33F2Av1 ↑
UGT33M1 ↑
UGT33F3 ↑
UGT33F1A ↑
UGT33F1Bb ↑
UGT33F1Ba ↑
UGT33F2B ↓
UGT33B4 ↓
UGT33J1 ↑
UGT33B5 ↓
UGT33B7A ↑
UGT33B7C ↓
UGT33B12b ↑
UGT33B12a ↑
UGT33B3 ↑
UGT33B11a ↑
UGT33B9A ↑
UGT33B11b ↑
UGT33B8 ↑
