# Supplementary material for: Coordinated P450–UGT detoxification contributes to furanocoumarin tolerance associated with host plant range divergence in Helicoverpa
Source: PLoS Biol. 2026 Sep 22;24(9):e3004013. doi: 10.1371/journal.pbio.3004013 (PMC13623160; doi:10.1371/journal.pbio.3004013)
Supplement: Supplementary file 7 [file pbio.3004013.s007.pdf]

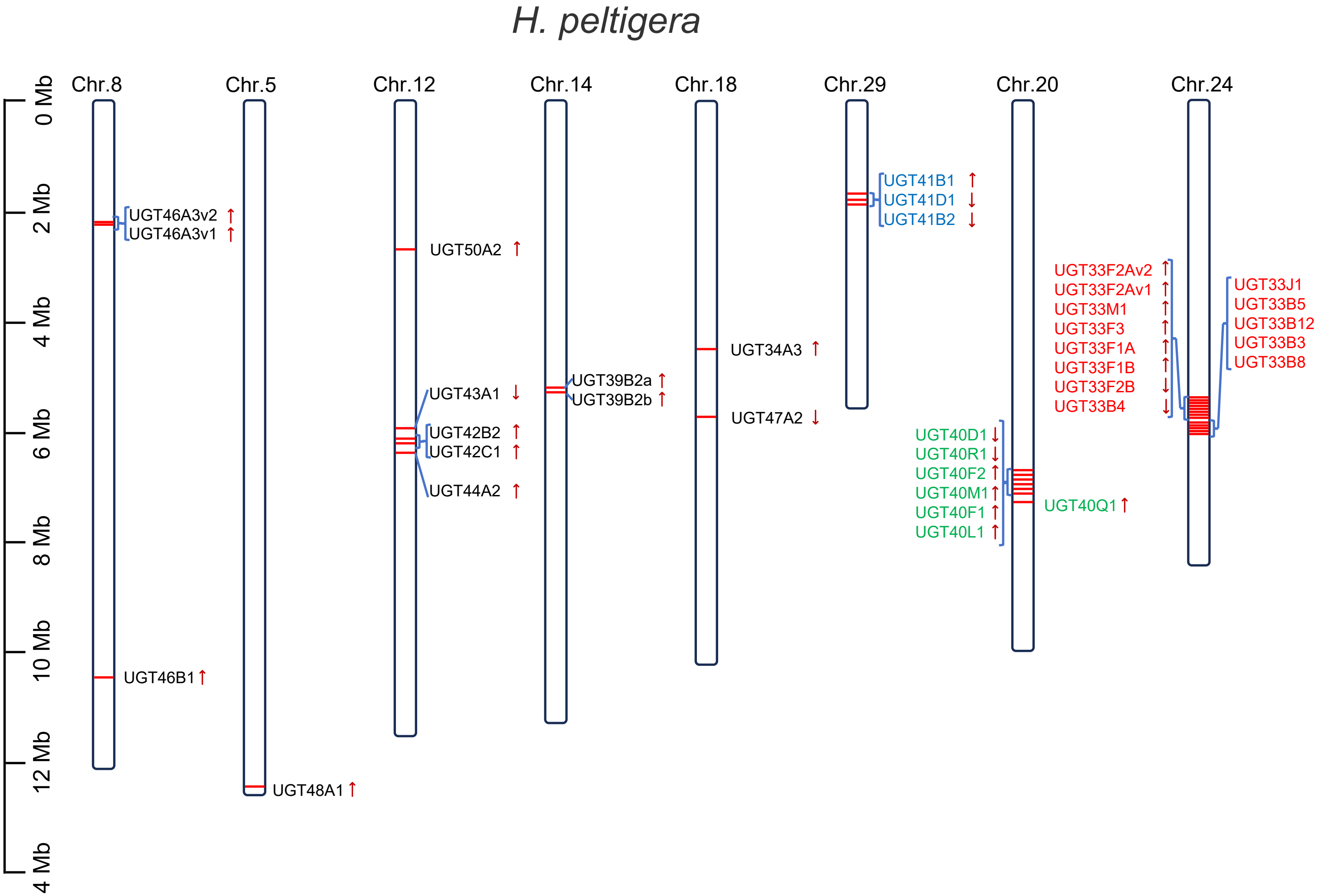

H. peltigera
0 Mb
2 Mb
4 Mb
6 Mb
8 Mb
10 Mb
12 Mb
14 Mb
Chr.8
Chr.5
Chr.12
Chr.14
Chr.18
Chr.29
Chr.20
Chr.24
UGT46A3v2 ↑
UGT46A3v1 ↑
UGT46B1 ↑
UGT48A1 ↑
UGT50A2 ↑
UGT43A1 ↓
UGT42B2 ↑
UGT42C1 ↑
UGT44A2 ↑
UGT39B2a ↑
UGT39B2b ↑
UGT34A3 ↑
UGT47A2 ↓
UGT41B1 ↑
UGT41D1 ↓
UGT41B2 ↓
UGT40D1 ↓
UGT40R1 ↓
UGT40F2 ↑
UGT40M1 ↑
UGT40F1 ↑
UGT40L1 ↑
UGT40Q1 ↑
UGT33F2Av2 ↑
UGT33F2Av1 ↑
UGT33M1 ↑
UGT33F3 ↑
UGT33F1A ↑
UGT33F1B ↑
UGT33F2B ↓
UGT33B4 ↓
UGT33J1 ↑
UGT33B5 ↓
UGT33B12 ↑
UGT33B3 ↑
UGT33B8 ↑
