# Supplementary material for: Coordinated P450–UGT detoxification contributes to furanocoumarin tolerance associated with host plant range divergence in Helicoverpa
Source: PLoS Biol. 2026 Sep 22;24(9):e3004013. doi: 10.1371/journal.pbio.3004013 (PMC13623160; doi:10.1371/journal.pbio.3004013)
Supplement: Supplementary file 8 [file pbio.3004013.s010.pdf]

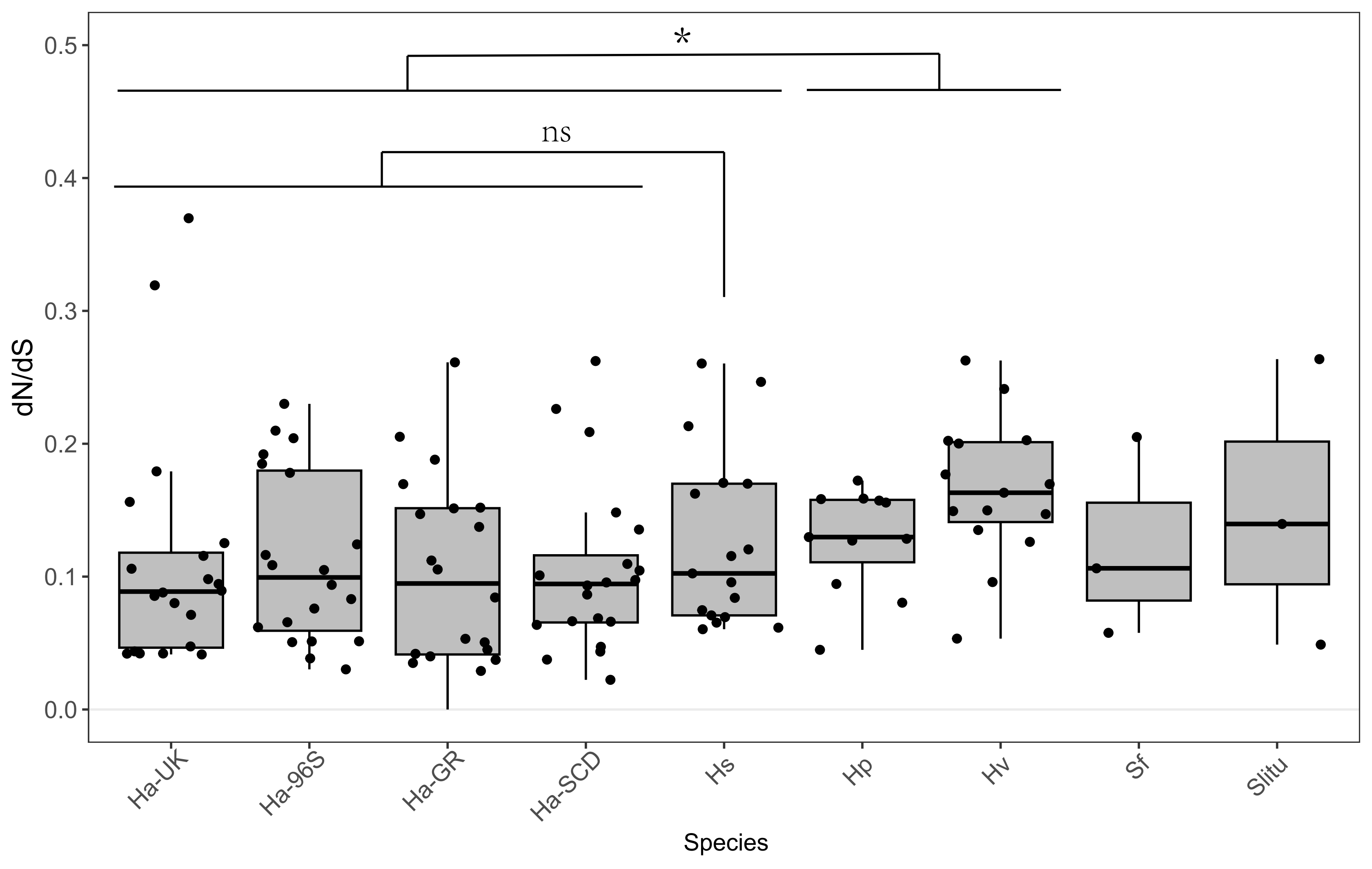

*
ns
0.5
0.4
0.3
0.2
0.1
0.0
dN/dS
Ha-UK
Ha-96S
Ha-GR
Ha-SCD
Hs
Hp
Hv
Sf
Slitu
Species
