# Supplementary material for: Coordinated P450–UGT detoxification contributes to furanocoumarin tolerance associated with host plant range divergence in Helicoverpa
Source: PLoS Biol. 2026 Sep 22;24(9):e3004013. doi: 10.1371/journal.pbio.3004013 (PMC13623160; doi:10.1371/journal.pbio.3004013)
Supplement: Supplementary file 9 [file pbio.3004013.s035.pdf]

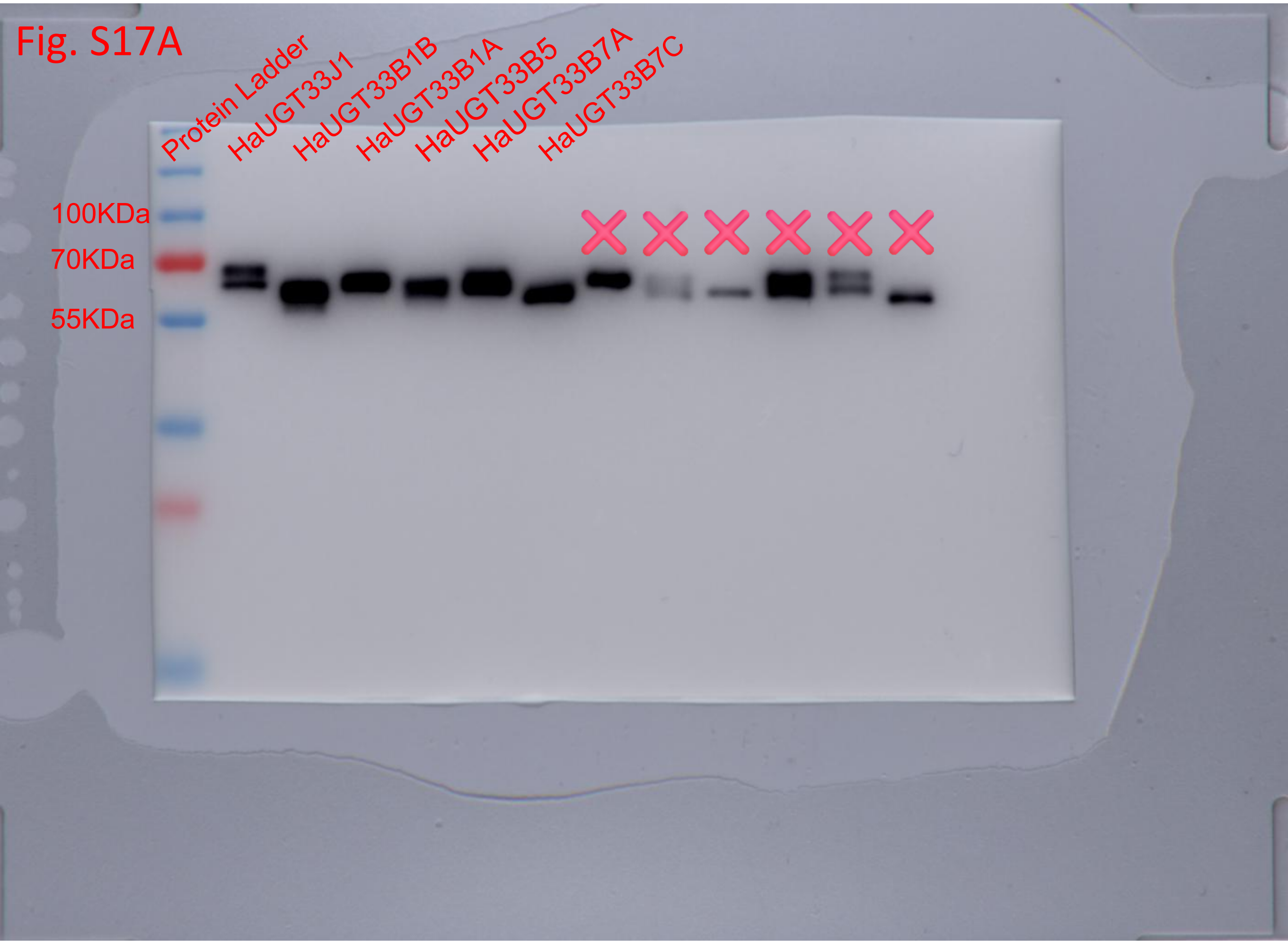
Fig. S17A
Protein Ladder
HaUGT33J1
HaUGT33B1B
HaUGT33B1A
HaUGT33B5
HaUGT33B7A
HaUGT33B7C
100KDa
70KDa
55KDa

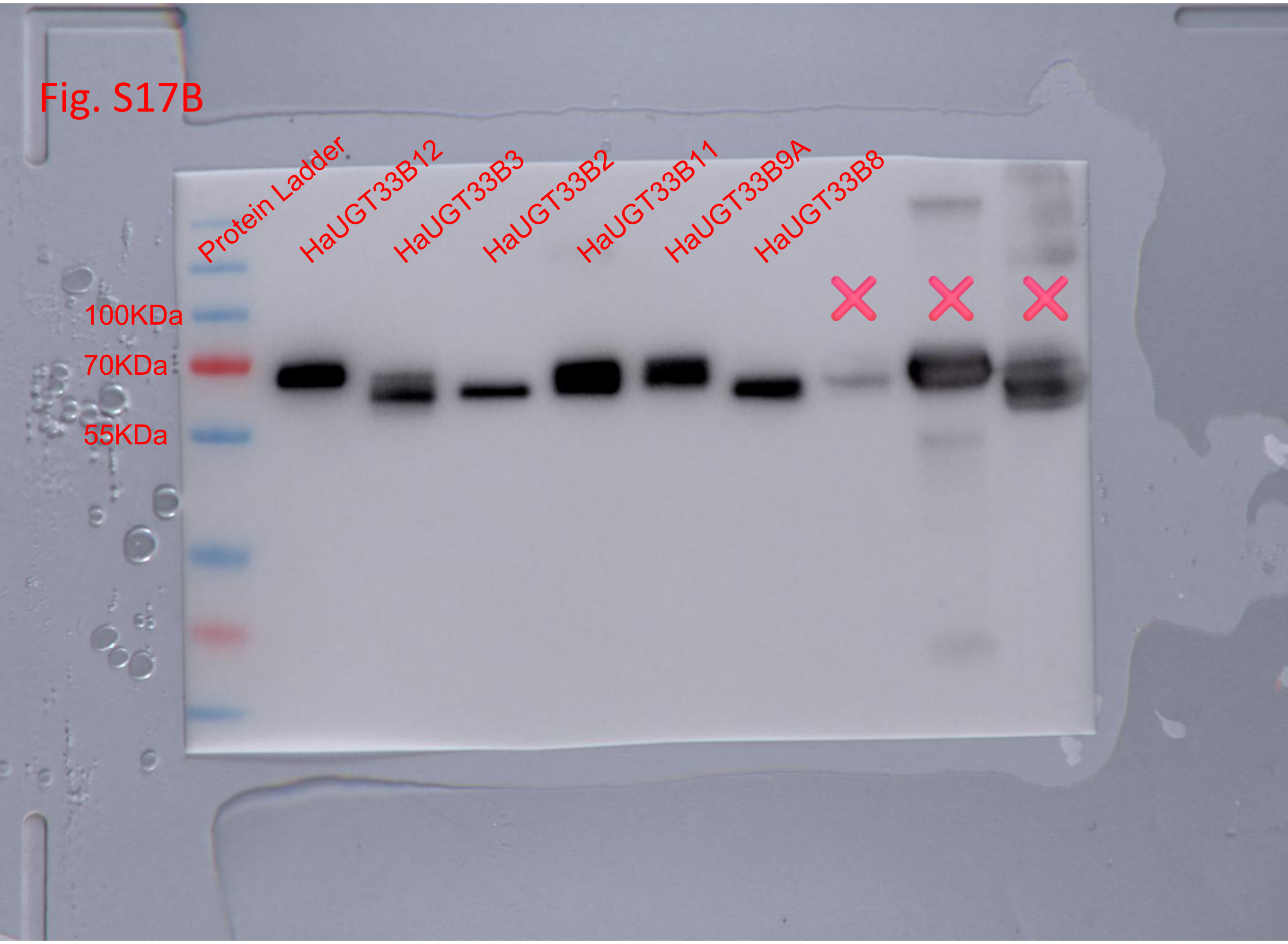
Fig. S17B
Protein Ladder
HaUGT33B12
HaUGT33B3
HaUGT33B2
HaUGT33B11
HaUGT33B9A
HaUGT33B8
100KDa
70KDa
55KDa

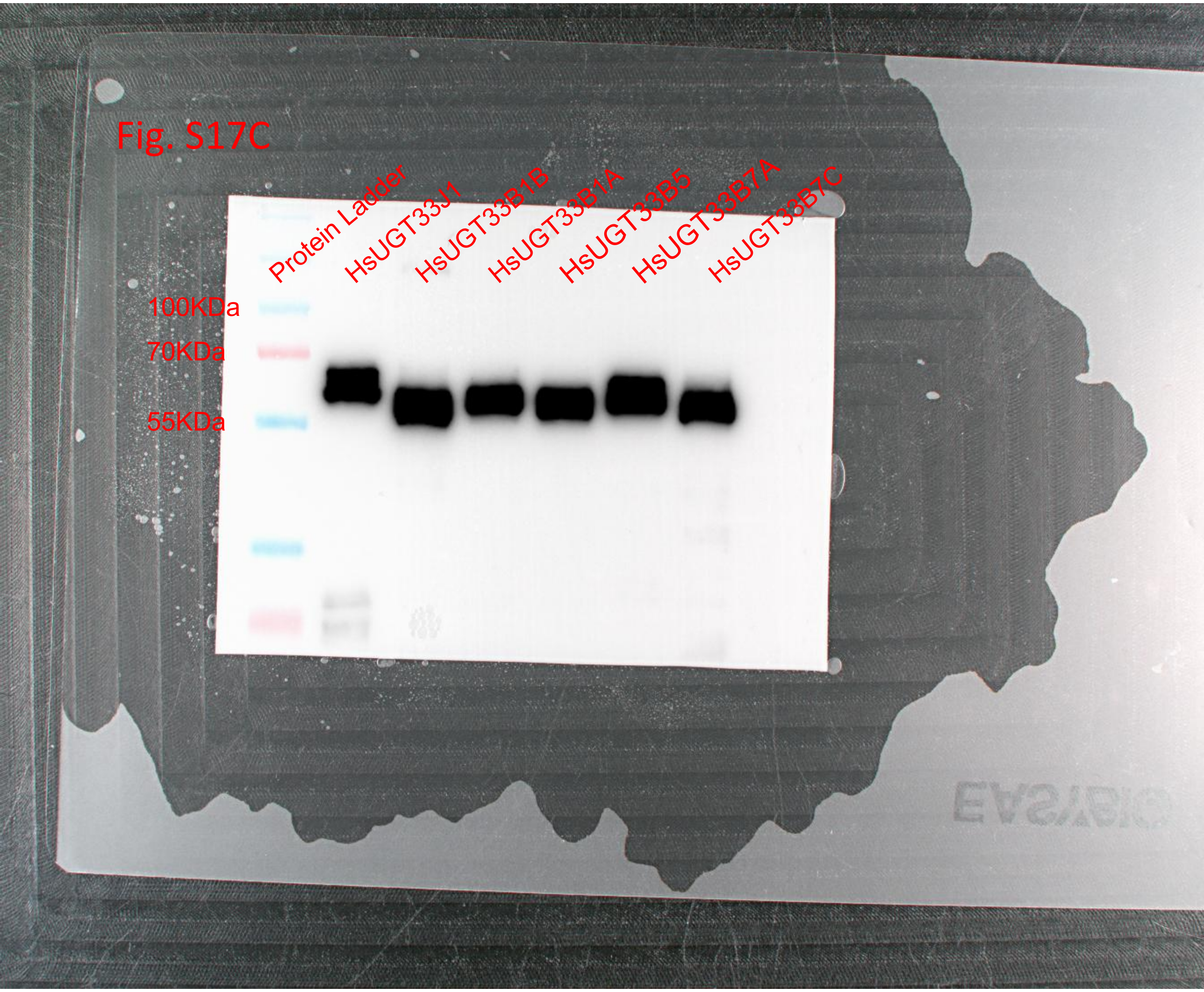

Fig. S17C
Protein Ladder
HsUGT33J1
HsUGT33B1B
HsUGT33B1A
HsUGT33B5
HsUGT33B7A
HsUGT33B7C
100KDa
70KDa
55KDa

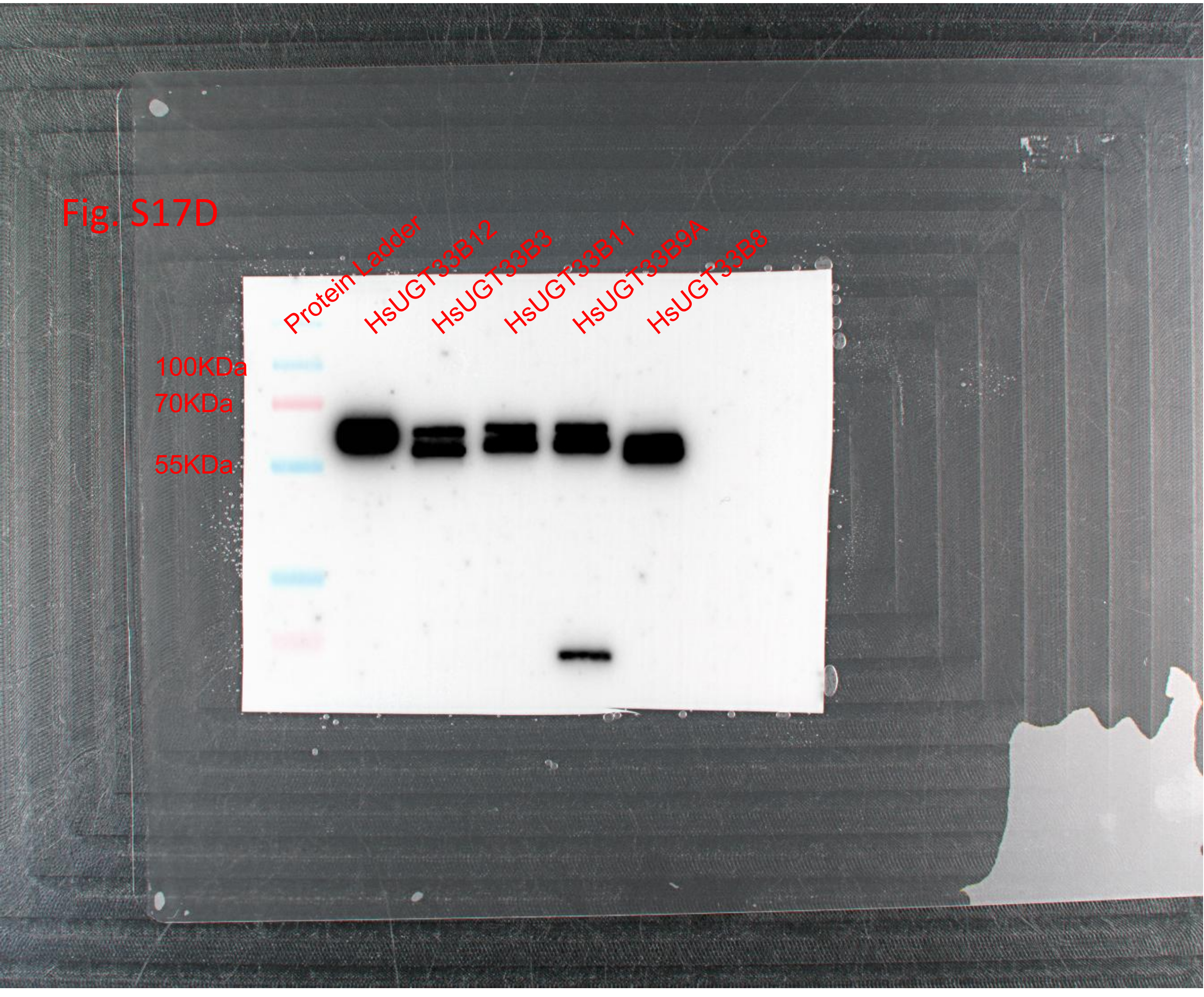
Fig. S17D
Protein Ladder
HsUGT33B12
HsUGT33B3
HsUGT33B11
HsUGT33B9A
HsUGT33B8
100KDa
70KDa
55KDa
