# Supplementary material for: Coordinated P450–UGT detoxification contributes to furanocoumarin tolerance associated with host plant range divergence in Helicoverpa
Source: PLoS Biol. 2026 Sep 22;24(9):e3004013. doi: 10.1371/journal.pbio.3004013 (PMC13623160; doi:10.1371/journal.pbio.3004013)

Fig. S15A

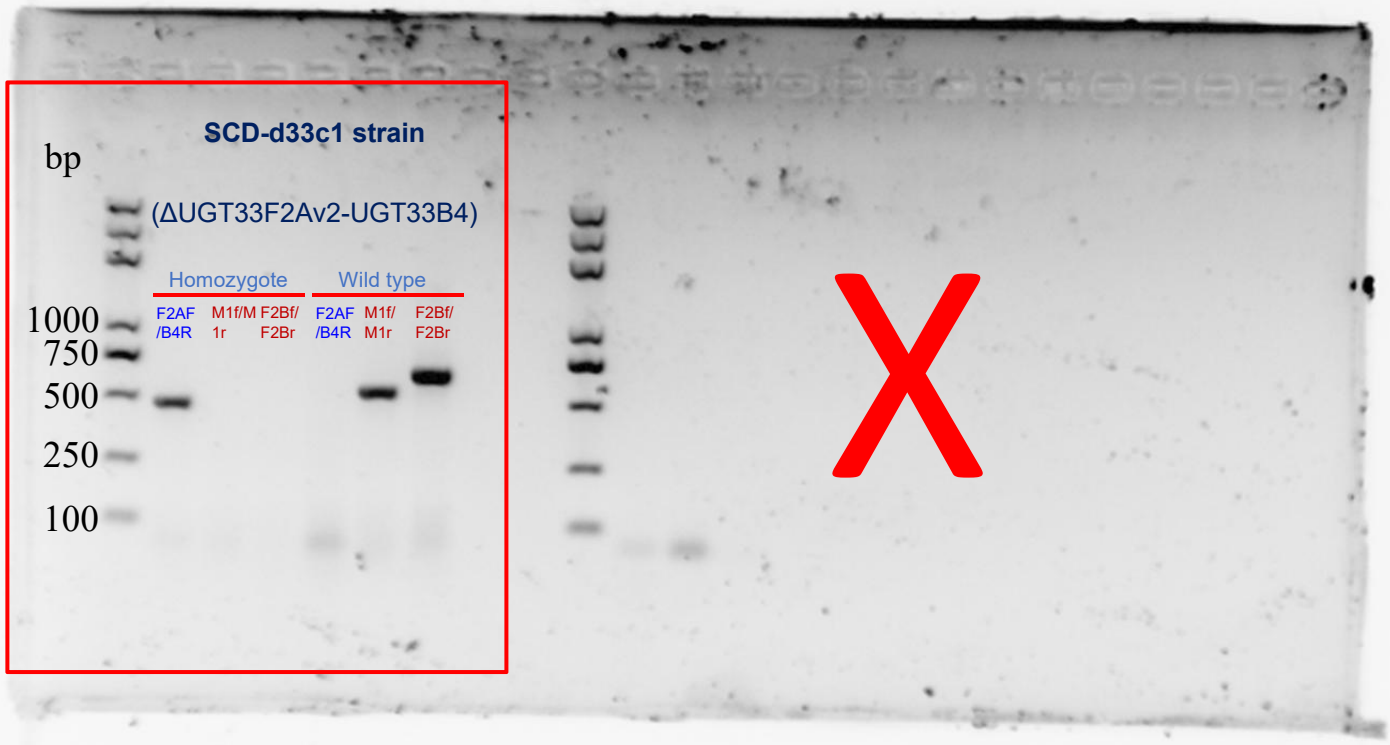

bp
SCD-d33c1 strain
(ΔUGT33F2Av2-UGT33B4)
Homozygote
Wild type
F2AF /B4R
M1f/M 1r
F2Bf/ F2Br
F2AF /B4R
M1f/ M1r
F2Bf/ F2Br
1000
750
500
250
100
X

Fig. S15B

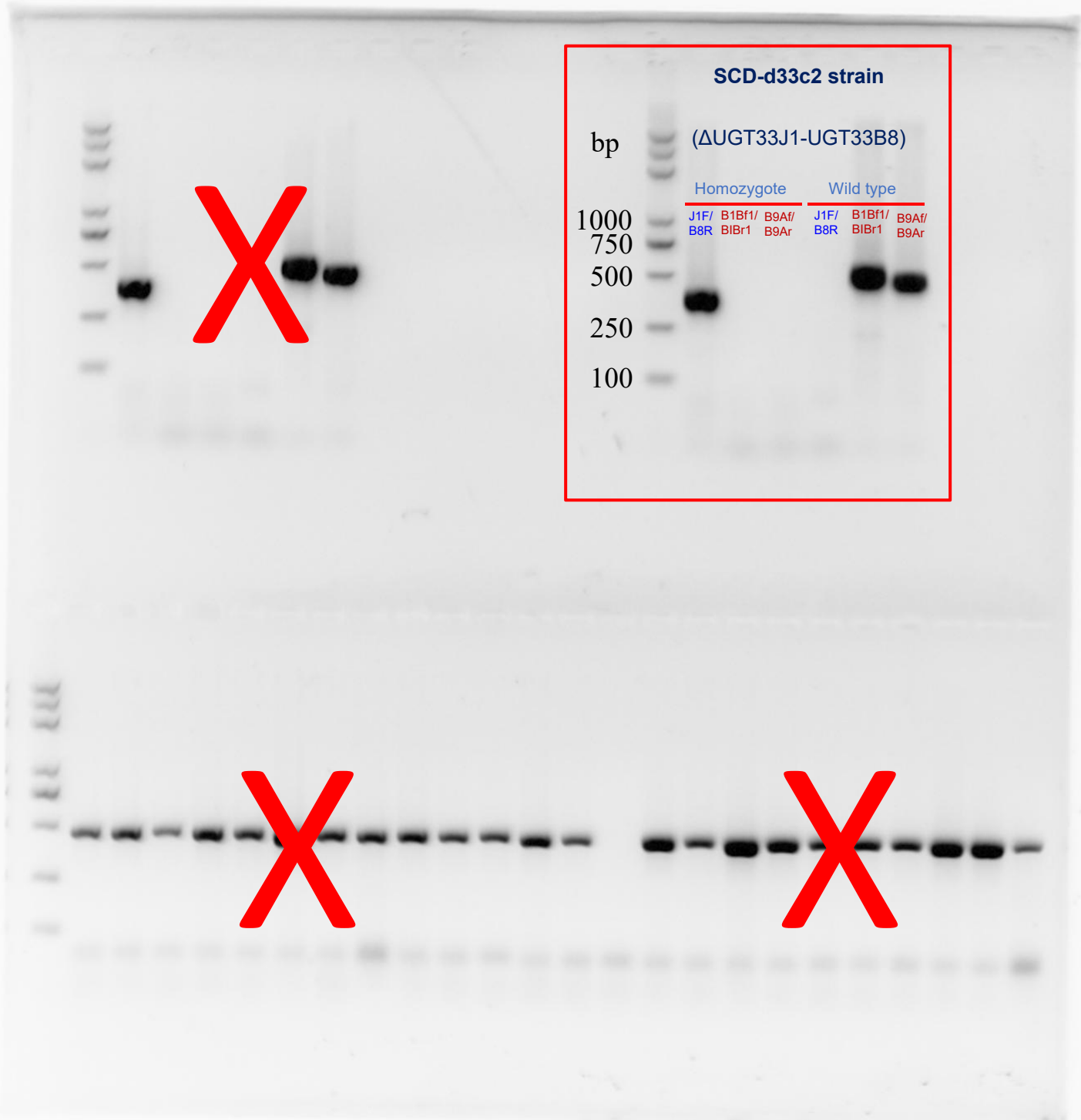

SCD-d33c2 strain
(ΔUGT33J1-UGT33B8)
bp
Homozygote
Wild type
1000
750
500
250
100
J1F/ B8R
B1Bf1/ BIBr1
B9Af/ B9Ar
J1F/ B8R
B1Bf1/ BIBr1
B9Af/ B9Ar

Fig. S15D

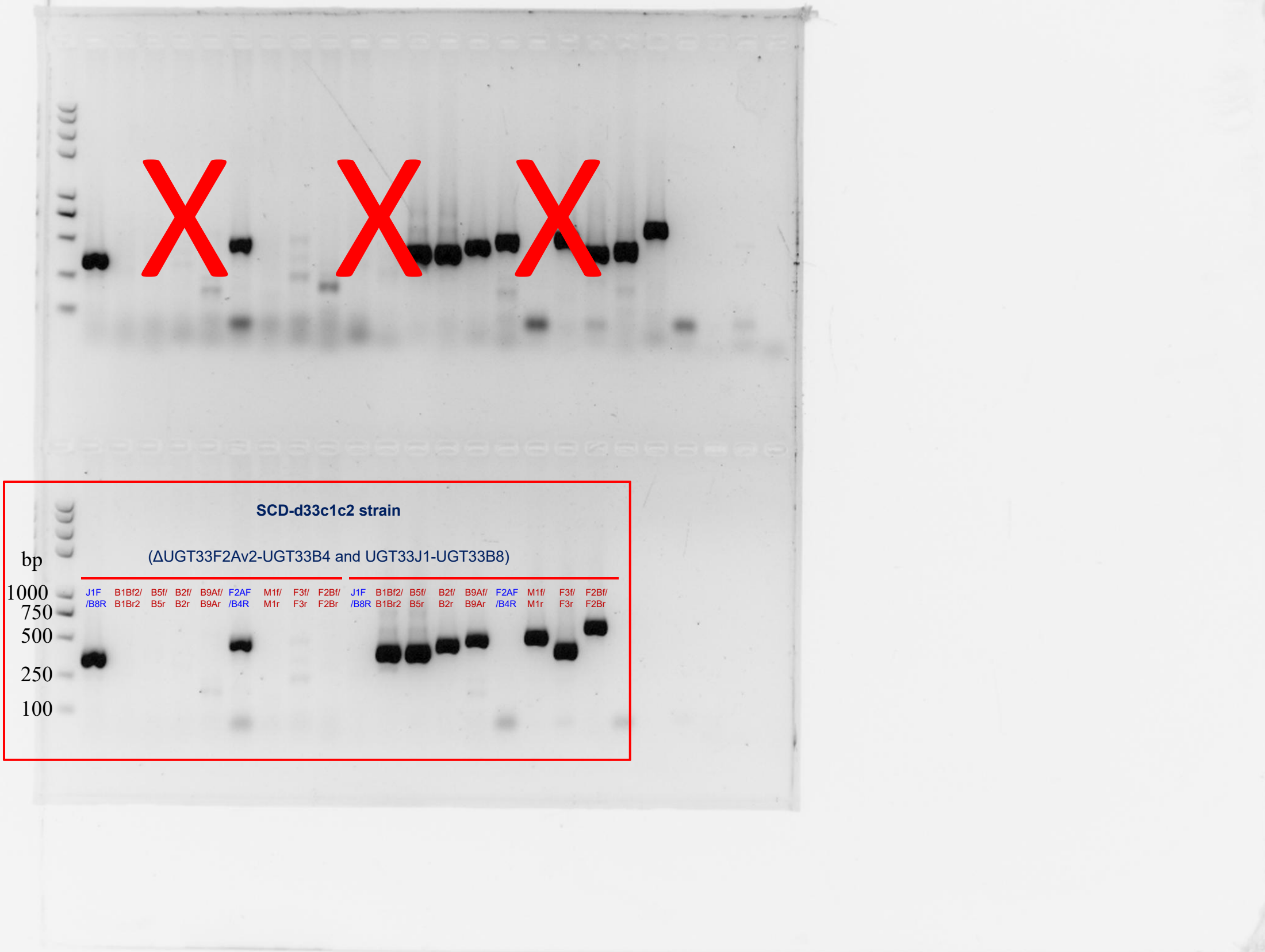
SCD-d33c1c2 strain
(ΔUGT33F2Av2-UGT33B4 and UGT33J1-UGT33B8)
bp
1000
750
500
250
100
J1F /B8R
B1Bf2/ B1Br2
B5f/ B5r
B2f/ B2r
B9Af/ B9Ar
F2AF /B4R
M1f/ M1r
F3f/ F3r
F2Bf/ F2Br
J1F /B8R
B1Bf2/ B1Br2
B5f/ B5r
B2f/ B2r
B9Af/ B9Ar
F2AF /B4R
M1f/ M1r
F3f/ F3r
F2Bf/ F2Br

Fig. S20B

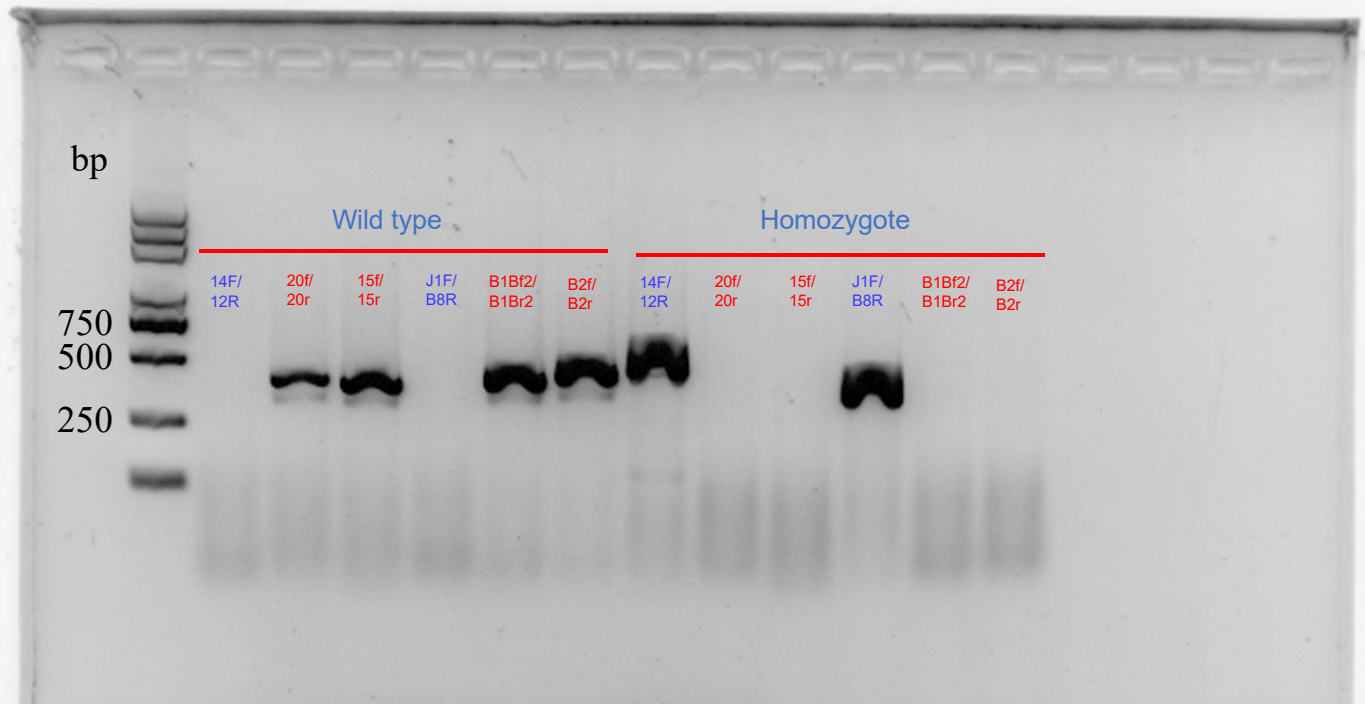

Fig. S21C

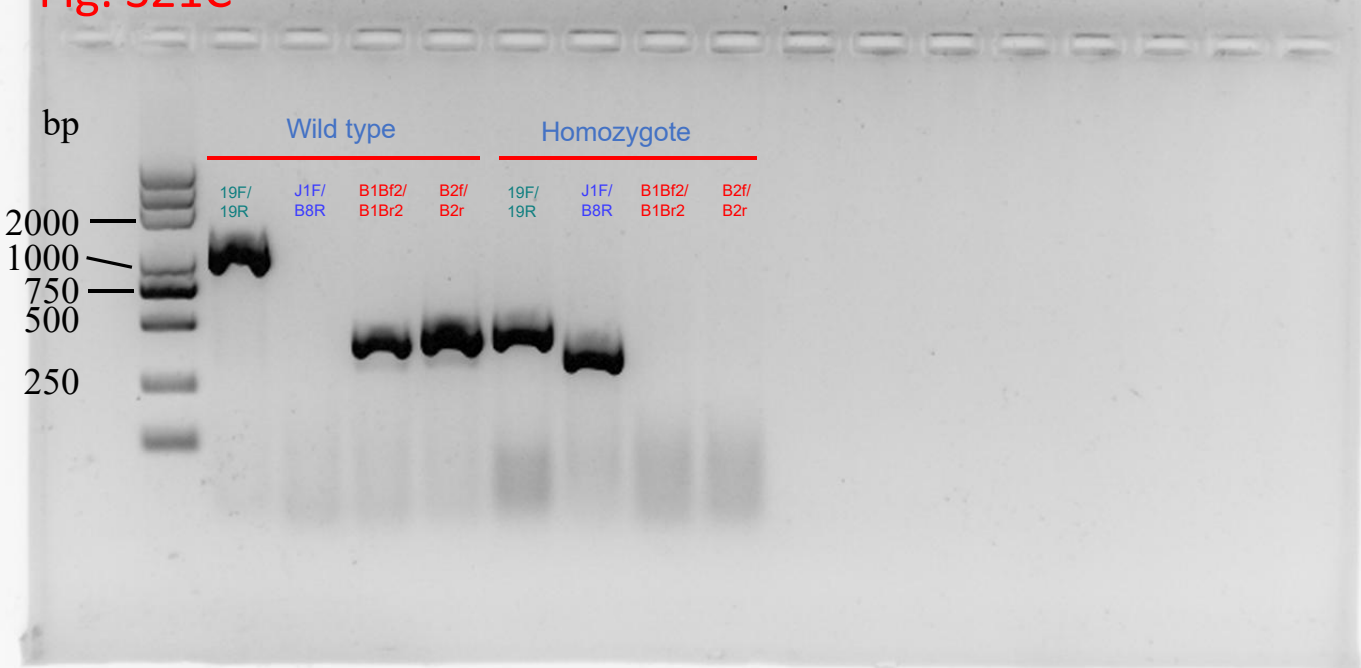

Supplement: Supplementary file 10 [file pbio.3004013.s036.pdf]
