# Supplementary material for: Coordinated P450–UGT detoxification contributes to furanocoumarin tolerance associated with host plant range divergence in Helicoverpa
Source: PLoS Biol. 2026 Sep 22;24(9):e3004013. doi: 10.1371/journal.pbio.3004013 (PMC13623160; doi:10.1371/journal.pbio.3004013)
Supplement: Supplementary file 11 [file pbio.3004013.s029.docx]

**S1 Table. Resequencing of the *H. armigera* SCD strain genome**. The sequencing depth across the entire chromosome 28 and the UGT gene clusters was determined using the inbuilt depth function of Samtools.

| Sample | depth(chr28) | depth(chr28:6550000-6880000) | depth(chr28:8820000-9080000) |
| --- | --- | --- | --- |
| SCD-1 | 23.0557 | 12.1408 | 14.1323 |
| SCD-2 | 26.9464 | 14.3274 | 16.4627 |
| SCD-3 | 24.2828 | 12.9319 | 14.9232 |
| SCD-4 | 24.6793 | 12.9124 | 15.2587 |
| SCD-5 | 23.6494 | 14.4939 | 12.8182 |
| SCD-6 | 22.962 | 14.1845 | 12.1938 |
| SCD-7 | 22.7987 | 16.6326 | 10.9987 |
| SCD-8 | 22.816 | 11.8606 | 13.4903 |
| SCD-9 | 21.6029 | 12.9267 | 11.314 |
| SCD-10 | 25.0103 | 13.1886 | 14.405 |
| SCD-pacbio | 105.404 | 64.7937 | 62.4927 |

Note: SCD1–10 represent the ten pupae subjected to resequencing, and SCD-pacbio corresponds to the individual used for the reference genome assembly.
