# Supplementary material for: Coordinated P450–UGT detoxification contributes to furanocoumarin tolerance associated with host plant range divergence in Helicoverpa
Source: PLoS Biol. 2026 Sep 22;24(9):e3004013. doi: 10.1371/journal.pbio.3004013 (PMC13623160; doi:10.1371/journal.pbio.3004013)
Supplement: Supplementary file 12 [file pbio.3004013.s030.docx]

**S2 Table. Sensitivity of newly-hatched larvae of different strains to xanthotoxin.**

| Strain | LC_50_  (mg/g) | 95% FL  (mg/g) | Slope ± SE | Toxicity ratio |
| --- | --- | --- | --- | --- |
| Ha-SCD | 5.80 | 5.36 - 6.19 | 6.50 ± 0.55 | 1.00 |
| SCD-d33c1 | 7.39 | 6.30 - 8.63 | 3.45 ± 0.45 | 0.78 |
| SCD-d33c2 | 2.88 | 2.40 - 3.26 | 4.53 ± 0.57 | 2.02 |
| SCD-d33c1c2 | 2.74 | 2.240 - 3.35 | 2.58 ± 0.16 | 2.12 |
| SCD-d6AE | 1.39 | 1.14 - 1.70 | 3.12 ± 0.23 | 4.18 |
| SCD-d6AE-d33c2 | 1.18 | 0.95 - 1.44 | 3.80 ± 0.34 | 4.93 |
| SCD-d6AE19-d33c2 | 1.02 | 0.79 - 1.33 | 2.32 ± 0.16 | 5.69 |
| Hs-XC | 0.50 | 0.43 - 0.57 | 4.50 ± 0.58 | 11.60 |

Note: LC_50_, lethal concentration that kills 50% of larvae; 95% FL, 95% fiducial limits of LC_50_; Toxicity ratio was calculated as LC_50_ of Ha-SCD strain/LC_50_ of knockout strain or Hs-XC.
