# Supplementary material for: Coordinated P450–UGT detoxification contributes to furanocoumarin tolerance associated with host plant range divergence in Helicoverpa
Source: PLoS Biol. 2026 Sep 22;24(9):e3004013. doi: 10.1371/journal.pbio.3004013 (PMC13623160; doi:10.1371/journal.pbio.3004013)
Supplement: Supplementary file 13 [file pbio.3004013.s031.docx]

**S3 Table. PCR primers used in this study.**

| Purpose | Primer name | Sequences (5'-3') |
| --- | --- | --- |
| Preparation of template DNAs for sgRNAs | sgRNA-33J1F | TAATACGACTCACTATAGTGATTCAAGTGAGTTCCTT |
|  | sgRNA-33J1R | TTCTAGCTCTAAAACAAGGAACTCACTTGAATCAC |
|  | sgRNA-33B8F | TAATACGACTCACTATAGAAATTCGATTTGATCATGT |
|  | sgRNA-33B8R | TTCTAGCTCTAAAACACATGATCAAATCGAATTTC |
|  | sgRNA-33F2Av2F | TAATACGACTCACTATAGATATAAGAAAATGCCAACGC |
|  | sgRNA-33F2Av2R | TTCTAGCTCTAAAACGCGTTGGCATTTTCTTATATC |
|  | sgRNA-33B4F | TAATACGACTCACTATAGAGGATAGCTTTCCAAATGA |
|  | sgRNA-33B4R | TTCTAGCTCTAAAACTCATTTGGAAAGCTATCCTC |
|  | sgRNA-6AE19F1 | TAATACGACTCACTATAGAGGGAGTCGACAGACTACTG |
|  | sgRNA-6AE19R1 | TTCTAGCTCTAAAACCAGTAGTCTGTCGACTCCCT |
|  | sgRNA-6AE19F2 | TAATACGACTCACTATAGTGCGGGTTACGAAACATCCG |
|  | sgRNA-6AE19R2 | TTCTAGCTCTAAAACCGGATGTTTCGTAACCCGCA |
| Detection of the UGT33 cluster 1 deletion | F2AF | TCAGGACCAAACCTGTCTCTG |
|  | B4R | GGATACAGCAAGGGATGCCA |
|  | M1f | GTCACCGTCATCACTCCAGA |
|  | M1r | ATTCAGCCAAAGGCGGTGTT |
|  | F3f | AGCTGTCATAGATGCCCAGT |
|  | F3r | GGAGGTACAGGTCGAATCCC |
|  | F2bf | ACCACAGATCCTGCATTTGGA |
|  | F2Br | GGTGCATTCCACCCATATACAC |
| Detection of the UGT33 cluster 2 deletion | J1F | GACACCCATGGCAGTTACGA |
|  | B8R | CCTGCATTGAGGGAAGGTCA |
|  | B1Bf1 | GGAGAAGCACCGGCTAATCT |
|  | B1Br1 | CTGGGAGGAACTGGACGATT |
|  | B1Bf2 | GTGTTCCGACCTCTTACGCA |
|  | B1Br2 | AGGATACAGCAAAGGGTGCC |
|  | B5f | CGAGGACATGAAGTCACGGT |
|  | B5r | GTCCCACTTGCTCAGGTTGT |
|  | B2f | AACGATGCGGCTCGAATACT |
|  | B2r | GGTGCCATGCCATTCCCATA |
|  | B9Af | CCCTGAAGGAGAAGCACCAG |
|  | B9Ar | CTAGCTCTGCTACAGACGGC |
| Detection of the *CYP6AE19* knockout | 19F | TTCTCGCGGTCTTAGTGACC |
|  | 19R | GTGTCAAGTTGCCTAGGACC |
| Detection of the CYP6AE cluster deletion | 14F | AAACCTTTCCCTGTCGGCAT |
|  | 12R | AGATGGCCAAATAGCCCGAG |
|  | 20f | ACACCATGGCACCAATCCAT |
|  | 20r | CAGATGTCTCGTAACCCGCA |
|  | 15f | GGCATTTTTAGCGCACGTCA |
|  | 15r | TTGCTAGCACTGGGTACAGC |
| Cloning the cDNA sequences of the UGTs for protein expression | HaUGT33J1-eF | TCACTAGTCGCGGCCGCACCATGGCTACACTTAACTACCT |
|  | HaUGT33J1-eR | TACCGCATGCCTCGAGTTAAGTACTTTTAAGTTTAGCTT |
|  | HaUGT33B1B-eF | TCACTAGTCGCGGCCGCACCATGATGTCGTTTTGTTTGTT |
|  | HaUGT33B1B-eR | TACCGCATGCCTCGAGCTAATTGTCTTTTAGCTTCTGTTTT |
|  | HaUGT33B1A-eF | TCACTAGTCGCGGCCGCACCATGATGTCGCTGGCCTTG |
|  | HaUGT33B1A-eR | TACCGCATGCCTCGAGTCAATTACCTTTTAACTTCTTCTGT |
|  | HaUGT33B5-eF | TCACTAGTCGCGGCCGCACCATGATGTCGCTTGCCT |
|  | HaUGT33B5-eR | TACCGCATGCCTCGAGTCAATTATCTTTTAGCTTCTTCTTT |
|  | HaUGT33B7A-eF | TCACTAGTCGCGGCCGCACCATGATATCGGTCGCT |
|  | HaUGT33B7A-eR | TACCGCATGCCTCGAGTCAATTACTTTTTATTTTCTCCTT |
|  | HaUGT33B7C-eF | TCACTAGTCGCGGCCGCACCATGAGGTCGCTCGCA |
|  | HaUGT33B7C-eR | TACCGCATGCCTCGAGTTAGTGATCTTTAAGCTTTCTCTGT |
|  | HaUGT33B12-eF | TCACTAGTCGCGGCCGCACCATGTCGCCTGTTCTACT |
|  | HaUGT33B12-eR | TACCGCATGCCTCGAGTCAGTTATCTTTAAGCTTCTTCAGT |
|  | HaUGT33B3-eF | TCACTAGTCGCGGCCGCACCATGATGTTGCCGTTTGTTTTACTCG |
|  | HaUGT33B3-eR | TACCGCATGCCTCGAGTTAATTGTCTTTCAACTTCTTATGATCT |
|  | HaUGT33B2-eF | TCACTAGTCGCGGCCGCACCATGTTATTAGCCTCAGT |
|  | HaUGT33B2-eR | TACCGCATGCCTCGAGTTAGTTGGCTTTAATTTTTTTATCA |
|  | HaUGT33B11-eF | TCACTAGTCGCGGCCGCACCATGTTTATTGCTTTGATTTTG |
|  | HaUGT33B11-eR | TACCGCATGCCTCGAGTCATTGTTTCTTAATTTTTTTAGGC |
|  | HaUGT33B9A-eF | TCACTAGTCGCGGCCGCACCATGTATATTTCTTTAGTTTT |
|  | HaUGT33B9A-eR | TACCGCATGCCTCGAGTTACGGTTTCTTTACTTTTTTATGT |
|  | HaUGT33B8-eF | TCACTAGTCGCGGCCGCACCATGTCTATTACTTTAATAA |
|  | HaUGT33B8-eR | TACCGCATGCCTCGAGTTATTGCTTCTTTATTTTTTTATGC |
|  | HsUGT33J1-eF | TCACTAGTCGCGGCCGCACCATGGCTACACTAAACTTCC |
|  | HsUGT33J1-eR | TACCGCATGCCTCGAGTTAAGTACTCTTAAGTTTGGCTTT |
|  | HsUGT33B1B-eF | TCACTAGTCGCGGCCGCACCATGATGTCGTTTTGTTTGTTT |
|  | HsUGT33B1B-eR | TACCGCATGCCTCGAGCTAATTGTGTTTTAGCTTCTGTTTT |
|  | HsUGT33B1A-eF | TCACTAGTCGCGGCCGCACCATGATGTCGCTGGCCT |
|  | HsUGT33B1A-eR | TACCGCATGCCTCGAGCTAATTGTCTTTTAGTTTCTGTTTT |
|  | HsUGT33B5-eF | TCACTAGTCGCGGCCGCACCATGATGTCGTTTGCCT |
|  | HsUGT33B5-eR | TACCGCATGCCTCGAGTCAATTGTCTTTTAGCTTCTTCTGT |
|  | HsUGT33B7A-eF | TCACTAGTCGCGGCCGCACCATGATATCGGTCGCT |
|  | HsUGT33B7A-eR | TACCGCATGCCTCGAGTCAATTACTTTTTATTTTCTCTTT |
|  | HsUGT33B7C-eF | TCACTAGTCGCGGCCGCACCATGAGGTCGCTCGCT |
|  | HsUGT33B7C-eR | TACCGCATGCCTCGAGCTAATGATCCTTAAGCTTTCTCTGT |
|  | HsUGT33B12-eF | TCACTAGTCGCGGCCGCACCATGTCGCCAGTTCTA |
|  | HsUGT33B12-eR | TACCGCATGCCTCGAGTTAATTATCTTTAAGCTTCTTCTGT |
|  | HsUGT33B3-eF | TCACTAGTCGCGGCCGCACCATGTTGCCGTTTGTTTTAC |
|  | HsUGT33B3-eR | TACCGCATGCCTCGAGTCAATTGTCTTTCAACTTCTTATGC |
|  | HsUGT33B11-eF | TCACTAGTCGCGGCCGCACCATGTTCAGTGCTTTG |
|  | HsUGT33B11-eR | TACCGCATGCCTCGAGTTATTGTTTCTTATTTTTTTTTG |
|  | HsUGT33B9A-eF | TCACTAGTCGCGGCCGCACCATGTCTATTTCTTTAGTT |
|  | HsUGT33B9A-eR | TACCGCATGCCTCGAGTTACGGTTTCTTTATTTTTTTTTGT |
|  | HsUGT33B8-eF | TCACTAGTCGCGGCCGCACCATGTCCATTACTTTA |
|  | HsUGT33B8-eR | TACCGCATGCCTCGAGTTATTGCTTCTTTATTTTTTTATGT |
|  | HsCYP6AE19-eF | TCACTAGTCGCGGCCGCACCATGAACGCCCTACTCG |
|  | HsCYP6AE19-eR | TACCGCATGCCTCGAGTCACGCTCGTACATATTTTCTAC |
|  | HsCPR-eF | TCACTAGTCGCGGCCGCACCATGTCAGACAGCGCACA |
|  | HsCPR-eR | TACCGCATGCCTCGAGTTAACTCCATACATCTGCTGAAT |
| Determination of UGT33 gene copy number | HaUGT33J1-cF | TGACCTTCCCTCAATGCAGG |
|  | HaUGT33J1-cR | AGGCAGTTCCTTGTTGGGTT |
|  | HaUGT33B5-cF | CATCCTTTGCTGTACCCCGT |
|  | HaUGT33B5-cR | ACTCCCCTTGTTGCATCACA |
|  | HaUGT33B3-cF | GCTGTTTGTCATGCAAGGAGG |
|  | HaUGT33B3-cR | GTTCCTCAGTCACCGTGTCA |
|  | HaUGT33M1-cF | AAGCCTGTCCGAGACCTGTA |
|  | HaUGT33M1-cR | AGCCAATTCAGCCAAAGGTG |
|  | para-F | CTTCAGATTTTCGGCCACCA |
|  | para-R | GTACTTTCGACGGTTGGCGT |
