# Supplementary material for: Coordinated P450–UGT detoxification contributes to furanocoumarin tolerance associated with host plant range divergence in Helicoverpa
Source: PLoS Biol. 2026 Sep 22;24(9):e3004013. doi: 10.1371/journal.pbio.3004013 (PMC13623160; doi:10.1371/journal.pbio.3004013)
Supplement: Supplementary file 14 [file pbio.3004013.s032.docx]

**S4 Table. Biological characteristics of the SCD-d33c2 strain and its parental Ha-SCD strain of *H. armigera*.**

| Biological characteristics | Ha-SCD | SCD‑d33c2 |
| --- | --- | --- |
| Larval duration (days) | 15.14 ± 0.05 | 15.11 ± 0.04^ns^ |
| Pupal duration (days) | 14.17 ± 0.28 | 13.60 ± 0.17^ns^ |
| Pupation rate (%) | 85.00 ± 3.00 | 76.50 ± 2.87^ns^ |
| Pupal weight (mg) | 374.66 ± 4.76 | 361.78 ± 3.50^ns^ |
| Emergence rate (%) | 87.19 ± 3.34 | 94.93 ± 1.60^ns^ |
| Average fecundity (eggs/female) | 1074.25 ± 87.54 | 1080.54 ± 156.56^ns^ |
| Average hatching rate (%) | 21.91 ± 5.16 | 33.79 ± 7.47^ns^ |
| Net reproductive rate (R₀) | 121.94 ± 39.13 | 194.13 ± 63.23^ns^ |

Note: Values are presented as mean ± SE. n = 3-4 biologically independent samples. Unpaired *t* tests were used for statistical comparisons (ns, not significant).
